# Coagulation induced by C3aR-dependent NETosis drives protumorigenic neutrophils during small intestinal tumorigenesis

**DOI:** 10.1038/ncomms11037

**Published:** 2016-03-21

**Authors:** Silvia Guglietta, Andrea Chiavelli, Elena Zagato, Carsten Krieg, Sara Gandini, Paola Simona Ravenda, Barbara Bazolli, Bao Lu, Giuseppe Penna, Maria Rescigno

**Affiliations:** 1 Department of Experimental Oncology, European Institute of Oncology, Via adamello, 16, I-20139 Milan, Italy; 2 Institute of Experimental Immunology, University of Zurich, CH-8057 Zurich, Switzerland; 3 Division of Epidemiology and Biostatistic, European Institute of Oncology, I-20141 Milan, Italy; 4 Gastrointestinal and Neuroendocrine Tumor Unit, European Institute of Oncology, I-20141 Milan, Italy; 5 Childrens' Hospital, Harvard Medical School, Boston, Massachussetts 02115, USA; 6 Department of Oncology and Haemato-Oncology, University of Milan, Milan 20139, Italy

## Abstract

Excessive activation of blood coagulation and neutrophil accumulation have been described in several human cancers. However, whether hypercoagulation and neutrophilia are linked and involved in cancer development is currently unknown. Here we show that spontaneous intestinal tumorigenesis correlates with the accumulation of low-density neutrophils with a pro-tumorigenic N2 phenotype and unprompted neutrophil extracellular traps (NET) formation. We find that increased circulating lipopolysaccharide induces upregulation of complement C3a receptor on neutrophils and activation of the complement cascade. This leads to NETosis, induction of coagulation and N2 polarization, which prompts tumorigenesis, showing a novel link between coagulation, neutrophilia and complement activation. Finally, in a cohort of patients with small but not large intestinal cancer, we find a correlation between neutrophilia and hypercoagulation. This study provides a mechanistic explanation for the tumour-promoting effects of hypercoagulation, which could be used as a new biomarker or as a therapeutic target.

## Abstract (web summary)

It is unclear whether cancer-related hypercoagulation and neutrophilia contribute to tumorigenesis. In this study, the authors find that activation of the complement cascade causes hypercoagulation that leads to polarization of neutrophils in a mouse model of intestinal cancer, and show that blocking complement activation can reduce tumour formation.

## Graphical abstract

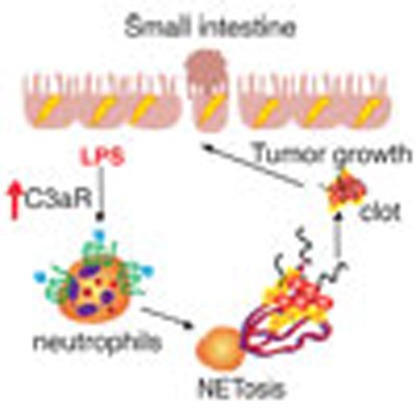

Clinical, histological and pharmacologic evidence supports a correlation between blood coagulation and tumorigenesis. In 1865, Trousseau noted that patients with visceral malignancies had an increase in thromboembolic diseases, which are the second most common cause of death in cancer patients^1^. Conversely, patients with venous thromboembolism (VTE) often have hidden visceral cancers. Post mortem histological evidence reveals that VTE occurs in ∼50% of cancer patients, and gastrointestinal and lung cancers possess the highest VTE rates^2^,^3^,^4^.

The occurrence of coagulation defects in cancer patients is a complex and poorly defined phenomenon. The involvement of oncogene activation in haemostatic defects has been reported in a spontaneous mouse model of sporadic hepatocarcinogenesis. In this model, the human oncogene MET was introduced in the somatic cells of the liver and this led to hypercoagulation and internal haemorrages^5^,^6^,^7^. Oncogene-induced coagulation led to fibrin deposition and hypoxia, which were exploited by tumour cells for their own growth and to foster vasculogenesis. Carcinoma-derived mucins also trigger the formation of microthrombi via a mechanism that involves selectins, platelets and neutrophil activation^6^. Activated oncogenes or inactivated tumour suppressor genes can also trigger tissue factor (TF) expression, resulting in increased coagulation, angiogenesis and development of more aggressive cancers^8^. A recent report has shown that a 50% reduction of prothrombin levels in mice heterozygous for a prothrombin-null allele (fII+/− mice) correlated with significantly fewer tumours in a model of inflammation-induced colorectal cancer (CRC)^7^. Although lacking a mechanistic explanation, this report provides a causative role for coagulation in intestinal cancer. Recent clinical studies demonstrated better cancer outcome and increased overall survival in patients who had been receiving anticoagulant treatments and in CRC patients given aspirin before cancer diagnosis, but it was not clear whether this was solely due to reduced VTE episodes^9^,^10^,^11^.

Neutrophilia has also been associated with poor prognosis in several epithelial malignancies^12^. To date, the role of neutrophils in cancer has been debated and controversial evidence has emerged from different studies. For instance, depletion of neutrophils was found to significantly reduce tumour growth^13^, whereas depletion of neutrophils at the time of T-cell priming resulted in ineffective control of syngeneic tumours in rats^14^. These contrasting results may be explained by the findings that the activity of neutrophils on tumour growth and progression could be dictated by context-dependent factors. Indeed, neutrophils can undergo polarization towards anti-tumorigenic (N1) or pro-tumorigenic (N2) phenotypes. Locally produced transforming growth factor (TGF)-β enhances tumour growth through the recruitment of N2 neutrophils in mouse models of mesothelioma and lung cancer^15^. Conversely, in a mouse model of breast cancer accumulating neutrophils efficiently prevented the development of lung metastases^16^.

Interestingly, neutrophils play a central role in thrombosis. For instance, a recent report has shown that neutrophils are the main leucocyte subset recruited within venous thrombi and are essential for the initiation and propagation of deep vein thrombosis^17^. Neutrophils can contribute to cancer-associated thrombosis by releasing neutrophil extracellular traps (NETs)^18^. In addition, a recent study described increased levels of neutrophil markers in the plasma of cancer patients undergoing acute thrombotic microangiopathies^19^. However, whether neutrophils and coagulation, by reciprocal interaction, could exert an effect on tumour growth is not known.

Here we demonstrate in a spontaneous small intestinal tumour model (APC^Min/+^ mice) that tumour development is associated with hypercoagulation and neutrophilia. Blood clots directly inhibit neutrophil effector functions. Hypercoagulation correlates with the appearance of low-density neutrophils (LDN), which display clear features of N2 neutrophils and spontaneously undergo NETosis. These effects are dependent on the engagement of the complement 3a receptor (C3aR), thus providing a mechanistic explanation for the tumour-promoting effects of blood coagulation.

## Results

### APC^Min/+^ mice develop haemostatic disorders

Intestinal cancers in patients are often diagnosed after anaemia or blood in the stool due to gastrointestinal bleeding^20^. Previously published reports and our own observations show that the development of polyps and the consequent ulcerations of the gastrointestinal tract in APC^Min/+^ mice induce progressive increase in blood loss^21^. As a consequence, APC^Min/+^ mice undergo severe anaemia as shown by the reduction in red blood cells (RBC) and haemoglobin (HGB) (Supplementary Fig. 1a,b). These findings, together with the observation that blood from APC^Min/+^ mice coagulated faster, prompted us to investigate the coagulation status in APC^Min/+^ mice during tumour development. First, we measured the prothrombin time (PT) in APC^Min/+^ and wild-type (WT) littermates. In WT littermates, we found an average of PT of 9.9 s (Supplementary Fig. 1c). However, starting at 12 weeks of age the majority of the APC^Min/+^ mice showed a reduction in the PT, which fell under the detection limit (<9.6 s). Next, we tested fibrinogen serum levels and platelet numbers. Although we observed no differences in the levels of soluble fibrinogen (Supplementary Fig. 1d) between APC^Min/+^ and WT littermates, we found decreased platelet number and increased platelet volume in APC^Min/+^ mice (Supplementary Fig. 1e,f). These results demonstrate that APC^Min/+^ mice undergo a progressive and chronic hyperactivation of the coagulation characterized by massive platelet recruitment.

### APC^Min/+^ mice show progressive accumulation of neutrophils

**Figure 1:**
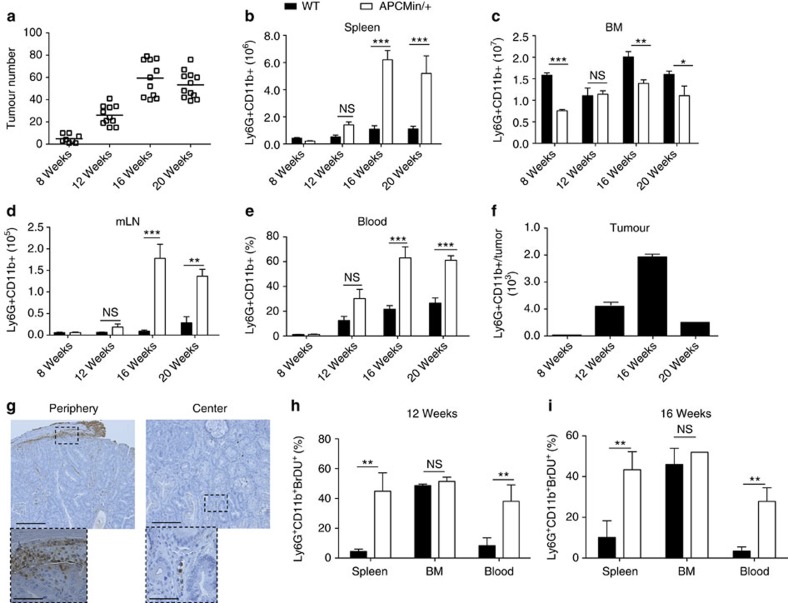
Progression of gastrointestinal tumorigenesis correlates with neutrophil accumulation in APC^Min/+^ mice. (**a**) APC^Min/+^ mice at 8, 12, 16 and 20 weeks of age were killed. Tumour number was evaluated by counting single polyps in small intestines and colons. (**b**–**f**) Single-cell suspensions were prepared from the spleen, BM, mLN, peripheral blood and intestinal tumours of APC^Min/+^ mice and WT littermates killed at indicated ages, and neutrophils were assessed using flow cytometry. The graphs show total numbers or frequencies of CD45.2+ Ly6G+ CD11b+ neutrophils in respective organs. Six to 8 mice per group were used for each time point. (**g**) Ly6G immunohistochemistry performed on a representative intestinal polyps of a 20-week-old APC^Min/+^ mouse. Top photos show the periphery and the central part of a polyp at × 10 original magnification (scale bar, 1 mm). Small boxes in the bottom show a 40-fold magnification of the dotted areas in the top photo (scale bar, 4 mm). (**h**,**i**) Twelve- and 16-week-old APC^Min/+^ mice and WT littermates were intraperitoneally (i.p.) injected with 1.5 mg of BrDU; the blood, spleen and BM were collected 24 h later and single-cell suspensions prepared. BrDU incorporation was evaluated in CD3- CD11b+Ly6G+ neutrophils by flow cytometry. Three mice were used for each time point. Results are representative of two independent experiments. Significance was calculated by using two-way analysis of variance (ANOVA) with Bonferroni post test (NS, not significant; *P<0.05, **P<0.01 and ***P<0.001). Bar graphs show means plus standard error of the mean (s.e.m.).

Intestinal tumorigenesis in APC^Min/+^ mice is associated with thymic atrophy, splenomegaly and reduction in T and B cells^22^. By performing a detailed analysis of the immune compartment in APC^Min/+^ mice, we found that the reduction in the lymphocytic population correlated with a dramatic increase in the number of neutrophils (defined as CD3^−^CD11b^+^Ly6G^+^), but we observed no difference in natural killer or dendritic cell populations (Fig. 1 and Supplementary Fig. 2). To understand whether the neutrophil increase was associated with the development of intestinal polyps, APC^Min/+^ mice and WT littermates were killed at different ages and tumour load was correlated with neutrophil numbers in the spleen, bone marrow (BM), mesenteric lymph nodes (mLN), peripheral blood and intestinal polyps (Fig. 1). As shown in Fig. 1b–e, at 8 weeks of age when polyps are virtually absent (Fig. 1a), no significant differences were observed in the number of neutrophils between APC^Min/+^ mice and WT littermates in the spleen, mLN and blood. In contrast, neutrophil numbers in the BM were significantly reduced in APC^Min/+^ mice (Fig. 1c).

At 12 weeks of age (when we observe a consistent growth of intestinal polyps), the neutrophil numbers increased in the spleen, blood and mLN of APC^Min/+^ mice, and peaked at 16 weeks of age when the development of polyps was maximal. These differences persisted through 20 weeks of age when the animals showed an exacerbation of the clinical signs^23^. As shown in Supplementary Fig. 3, in peripheral blood of 16- and 20-week-old APC^Min/+^ mice absolute numbers of neutrophils were higher as compared with that of WT littermates, indicating that the increased percentages were not a consequence of a reduction in other cell populations. By examining neutrophil numbers in intestinal polyps, we observed a characteristic bell-shaped curve with a peak at 16 weeks of age (Fig. 1f).

It has been recently shown that during tumour progression, tumour-associated neutrophils (TANs) become progressively able to migrate towards the tumour centre^24^. To assess the localization of TANs in this model, we performed immunohistochemistry on polyps isolated from small intestine of APC^Min/+^ mice at different ages. As shown in Fig. 1g, Ly6G^+^ TANs were found almost exclusively at the periphery of polyps, even in aged mice. This finding suggests that the polyps of APC^Min/+^ mice mirror the phenotype of early-stage tumours, consistent with their inability to become invasive.

Subsequently, we investigated the dynamics of neutrophil generation in WT and APC^Min/+^ mice during tumour development by using a 24-h 5-bromodeoxyuridine (BrdU) pulse to label the highly proliferative granulocytic compartment. Analysis by flow cytometry showed a significant increase in BrDU^+^ neutrophils in the blood and spleen but not in the BM of 12- to 16-week-old APC^Min/+^ mice, as compared with WT mice (Fig. 1h,i), suggesting that these neutrophils had recently proliferated and entered the circulation from the BM.

### Neutrophil increase depends on non-haematopoietic factors

**Figure 2:**
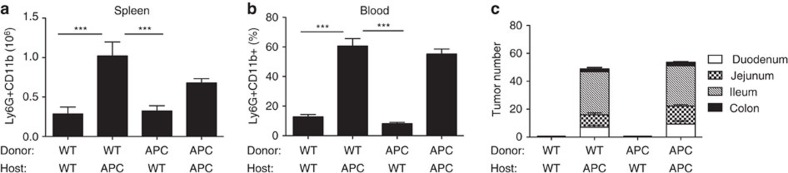
Neutrophil accumulation and tumour progression depend on haematopoietic extrinsic factors. (**a**,**b**) Seven-week-old APC^Min/+^ and WT host mice were lethally irradiated and reconstituted with Lin− BM cells from APC^Min/+^ or WT mice. Chimeric mice were killed at 20 weeks of age. Single-cell suspensions were prepared from (**a**) the spleen and (**b**) peripheral blood, and neutrophils assessed by flow cytometry. Shown are mean total neutrophil numbers plus s.e.m. in the spleen and frequencies in the blood. (**c**) Tumour counts in different regions of the small intestine and the colon of chimeric mice at 20 weeks of age. Results are pooled from two independent experiments (*n*=9 mice per group). Significance was calculated by using one-way analysis of variance (ANOVA) with Bonferroni post test (***P<0.001).

All haematopoietic stem cells express the Apc messenger RNA and Apc mutant cells are not able to maintain normal haematopoiesis^25^. Therefore, we generated BM chimeras, to distinguish whether the defects in the neutrophil compartment and in coagulation are intrinsic to the haematopoietic system. Lethally irradiated 7-week-old APC^Min/+^ or WT (CD45.1) hosts were transplanted with lineage negative (Lin−: CD3, CD4, CD8, CD19, Ter119 and Gr-1) precursors purified from WT (CD45.1) or APC^Min/+^ mice. One month after transplantation, we observed successful reconstitution in all groups of mice (Supplementary Fig. 4). Chimeric animals were killed 13 weeks after reconstitution and neutrophil numbers in the spleen and blood, as well as in tumours, were assessed. As shown in Fig. 2a,b, host APC^Min/+^ mice transplanted with WT or with APC^Min/+^ Lin− cells showed a clear increase in neutrophil numbers in the spleen and in the blood. In addition, only APC^Min/+^ hosts developed polyps. The distribution of polyps along the intestine did not change and they were predominant in the small intestine and, in particular, in the ileum, with only sporadic polyps in the colon, similar to non-chimeric APC^Min/+^ mice (Fig. 2c and also see ref. 26). Altogether, these results demonstrate that increased neutrophil numbers and intestinal tumorigenesis are dependent on non-haematopoietic factors.

### LDNs in the blood of aged APC^Min/+^ mice

**Figure 3:**
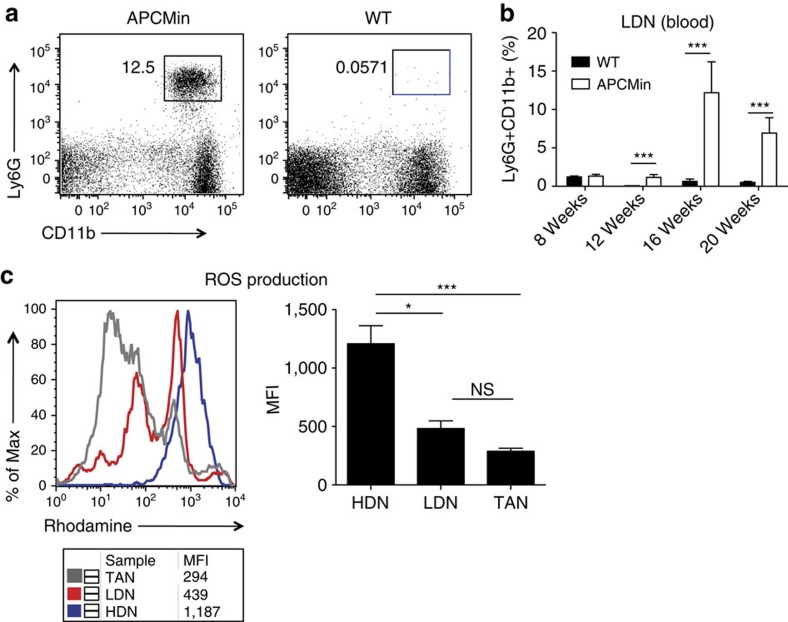
Accumulation and reduced function of LDNs in the peripheral blood of tumour-bearing APC^Min/+^ mice. (**a**) Blood was collected by heart puncture from 16- to 20-week-old APC^Min/+^ mice and WT littermates, and separated by density gradient centrifugation using Ficoll. Cells from the low-density fraction were collected and stained with α-CD45.2, α-CD11b and α-Ly6G antibodies. Shown are representative plots of LDNs found in the blood of 16-week-old APC^Min/+^ mice and WT littermates. (**b**) Statistic of the accumulation of LDNs in the peripheral blood of APC^Min/+^ mice. (**c**) LDNs and HDNs obtained by separating the blood on Ficoll gradient and FACS-sorted TANs were stimulated with 30 ng ml^−1^ PMA. ROS production was evaluated by flow cytometry through measuring oxidation of 1,2,3-dihydrorhodamine to rhodamine. Histograms show rhodamine+ Ly6G+ neutrophils and graphs summarize the statistic of mean fluorescence intensity (MFI) of the indicated cell populations. Results are representative of four independent experiments. Significance was calculated by using two-way analysis of variance (ANOVA) with Bonferroni post test (NS, not significant; *P<0.05 and ***P<0.001). 5 mice/group were used for the experiments in **a** and **b** respectively. In panel **c** 4 mice/group were used. Bar graphs show mean plus s.e.m.

Studies describing the role of neutrophils in mouse models of cancer usually focus on neutrophils present in the tumour microenvironment or isolated from whole blood using magnetic beads. To our surprise, we found that during intestinal tumorigenesis in APC^Min/+^ mice a sizable proportion of neutrophils (CD3^−^CD11^+^Ly6G^+^)—hereafter called LDNs (Fig. 3a)—sedimented in the mononuclear cell fraction. This finding is in agreement with a recent report describing the appearance of a low-density population of neutrophils with immunosuppressive function in a mouse model of 4T1 mammary tumour^27^. As shown in Fig. 3b, although WT littermates barely showed LDN at all analysed time points, APC^Min/+^ mice showed a dramatic increase in LDNs, reaching a maximum at 16 weeks of age.

Subsequently, we examined whether LDN showed different effector functions as compared with their high-density (HDN) counterparts or TANs. The best-characterized neutrophil effector function is their ability to produce reactive oxygen species (ROS); through this, they exert antimicrobial activity and exhibit cytotoxicity towards tumour cells, ultimately suppressing metastasis^16^,^28^,^29^. To this aim, LDNs, HDNs and TANs were purified from 16-week-old APC^Min/+^ mice. Subsequently, ROS production was assessed by dihydrorhodamin oxidation (Fig. 3c). HDNs produced significantly higher amounts of ROS as compared with LDNs and TANs, with LDNs being capable of producing intermediate levels of ROS.

These results show that in the APC^Min/+^ model, intestinal tumorigenesis correlates with a progressive accumulation of LDNs with reduced effector functions.

### Blood clots reduce neutrophil function inducing N2 phenotype

Having observed a coagulation defect and reduced effector functions of neutrophils in APC^Min/+^ mice, we investigated whether the increased coagulation could dampen innate immune responses and modulate neutrophil function.

**Figure 4:**
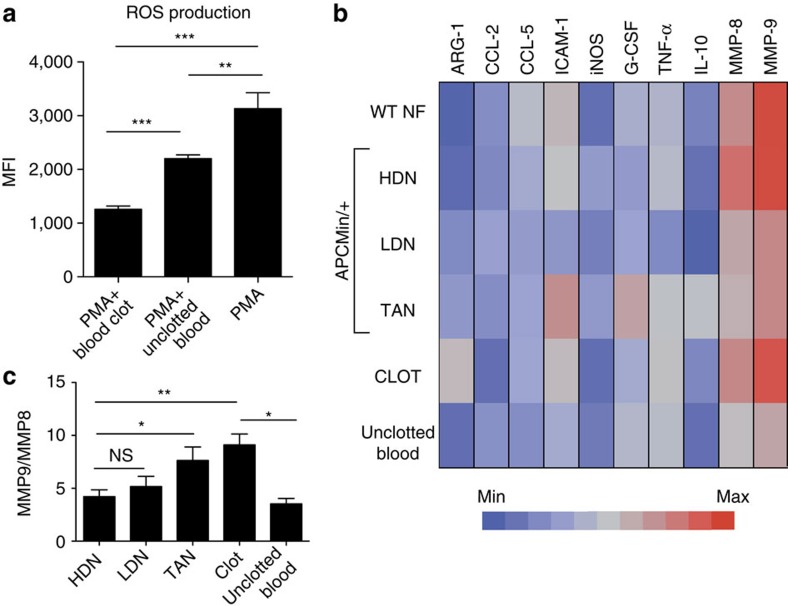
*In-vitro*-generated blood clots induce protumorigenic N2 neutrophils. (**a**) Neutrophils were purified from the BM of 12-week-old WT mice and left unstimulated, stimulated for 30 min with 10ng ml^−1^ PMA or 10 ng ml^−1^ PMA together with a blood clot or anticoagulant-treated blood from a 16-week-old APC^Min/+^ mouse. ROS production was evaluated by flow cytometry. The bar graph summarizes the statistic of mean fluorescence intensity plus s.e.m. (MFI): under the indicated conditions. Results are representative of five independent experiments. Significance was calculated by using an unpaired *t*-test (**P<0.01 and ***P<0.001). (**b**) Neutrophils were purified by Ficoll density centrifugation from the low- and high-density fractions of peripheral blood from 16- to 20-week-old WT and APC^Min/+^ mice, sorted from polyps of APC^Min/+^ mice or purified from the BM of WT mice. Purified neutrophils were subsequently lysed, RNA extracted, cDNA synthesized and used to perform real-time PCR. BM-derived neutrophils were stimulated for 12 h with blood clot or unclotted blood from APC^Min/+^ mice before RNA extraction. The expression of the examined genes was compared with the housekeeping *rpl32*. The expression profile of the different neutrophil populations is shown in a heat map generated with JMP 9.0. (**c**) MMP9/MMP8 ratio in the different populations of neutrophils of APC^Min/+^ mice. Results are representative of three independent experiments and are shown as mean plus s.e.m. Significance was calculated by using one-way analysis of variance (ANOVA) with Bonferroni post test (NS, not significant; *P<0.05).

We purified naive BM neutrophils from WT mice and treated them *in vitro* with blood clots generated from APC^Min/+^ mice or with equal amounts of anticoagulant-treated blood (unclotted blood). We then stimulated the neutrophils with phorbol myristate acetate (PMA) and measured ROS production as a readout of neutrophil functionality. As shown in Fig. 4a, blood thrombi were able to significantly reduce PMA-induced ROS production in neutrophils and this effect was significantly more pronounced as compared with anticoagulant-treated blood. Neutrophils have been characterized as highly plastic cells and it has been shown that they can polarize towards N1 or N2 phenotypes^15^. Therefore, we performed a gene expression analysis on *in-vitro*-stimulated or *ex-vivo*-isolated neutrophils. We collected HDNs, LDNs and TANs from APC^Min/+^ mice, whereas, owing to the very limited amount of LDNs, only HDNs were purified from WT mice. We compared the phenotype of the isolated neutrophils with that of BM neutrophils stimulated *in vitro* with blood clots or anticoagulant-treated blood from APC^Min/+^ mice. The heat map in Fig. 4b shows the gene expression profile of the different neutrophil populations obtained by performing real-time PCR for the displayed factors. Interestingly, LDNs, TANs and clot-treated neutrophils showed a more similar profile than the HDNs or unmanipulated WT HDNs. Consistent with a more ‘transitional' phenotype, LDNs had an HDN and TAN intermediate profile.

Interestingly, TANs were found to express the highest level of *g-csf*, which could explain the extensive recruitment of neutrophils into the circulation and subsequently into tumours (Fig. 4b). Finally, in LDNs, TANs and clot-treated neutrophils from APC^Min/+^ mice, we detected an increased ratio of the protumour metalloprotease *mmp-9* versus the antitumour *mmp-8*, as compared with that in HDNs (Fig. 4c). In addition, LDNs, TANs and clot-treated neutrophils shared features of N2 neutrophils; they all expressed higher levels of Arginase (*arg-1*), which has been described to inhibit adaptive immune responses and to characterize N2 neutrophils^15^. High levels of arginase mainly correlated with reduced levels of inducible nitric oxide synthase (*iNos*), according to the reciprocal regulation of the *arg-1* and *iNos* pathways, which both use L-arginine as a substrate^30^. To ensure that arginase was effectively derived from blood clot-activated neutrophils, we analysed *arg-1* expression in cells spontaneously released by the blood clots during culture. As shown in Supplementary Fig. 5a, *arg-1* in blood clot-stimulated neutrophils was significantly higher as compared with that of cells released from the blood clot. In addition, we also observed that blood clots derived from APC^Min/+^ mice induced higher *arg-1* expression in neutrophils as compared with the blood clots from WT mice. To elucidate the mechanism responsible for the immunomodulatory effect of blood clots, we assessed *arg-1* induction in neutrophils in a contact-dependent and -independent manner. To address this, neutrophils were cultured directly with blood clots or separated from blood clots by using a 0.4-μm transwell system. As shown in Supplementary Fig. 5b, although maximal *arg-1* induction was achieved when neutrophils were in contact with blood clots, we observed some induction of *arg-1* also in the absence of blood clot contact, suggesting that soluble factors might also play a role. As TGF-β1 has been shown to induce N2 neutrophils in the tumour microenvironment^15^ we investigated its possible role in our system. Interestingly, we found that blood clots of APC^Min/+^ mice produced significantly higher amounts of TGF-β1 as compared with WT blood clots (Supplementary Fig. 5c), possibly explaining the higher immunomodulatory capacity of APC^Min/+^ blood clots.

Altogether, these results show that the increased number of neutrophils in APC^Min/+^ mice are functionally skewed towards an N2 phenotype and LDNs are probably transitional N2 neutrophils that will develop into TANs.

### LMWH reduces intestinal tumors and LDN in APC^Min/*+*^ mice

Having observed a correlation between coagulation and tumour development, we assessed whether we could interfere with tumorigenesis by providing anticoagulants used in the clinics (low-molecular-weight heparin (LMWH) and warfarin). LMWHs are derived from unfractionated heparin by chemical or enzymatic depolymerization, yielding fragments of one-third of the size of heparin. LMWHs are the recommended anticoagulants in cancer patients, owing to their reduced side effects^31^,^32^. However, some studies report inhibition of selectin-mediated migration of immune cells by heparins^33^. Thus, we first ensured that LMWHs did not affect neutrophil migration. We found that LMWHs did not inhibit thioglycollate-induced neutrophil recruitment (Supplementary Fig. 6). Similarly, LMWHs have reduced effects on angiogenesis^34^ and, although controversial, it has been shown that the use of LMWHs in patients with inflammatory bowel diseases did not have a major impact on inflammatory parameters^35^.

Next, we examined whether anticoagulants could revert the hypercoagulation in APC^Min/+^ mice and have an impact on tumour burden. We treated 7-week-old APC^Min/+^ mice with the LMWH enoxaparin (Clexane) or warfarin, an inhibitor of vitamin K recycling often used in cancer patients to prevent cancer-associated VTE^36^. Because of the short half-life (4,5 h) of enoxaparin, we carried out the treatment by using subcutaneously (s.c.) implanted osmotic pumps, therefore ensuring a constant supply of anticoagulant to the animals. Animals were monitored for blood coagulation time, to confirm treatment efficacy.

After 12 weeks of treatment, mice were killed, peripheral blood was collected, tumour number assessed and neutrophils were purified, both from peripheral blood and intestinal polyps.

**Figure 5:**
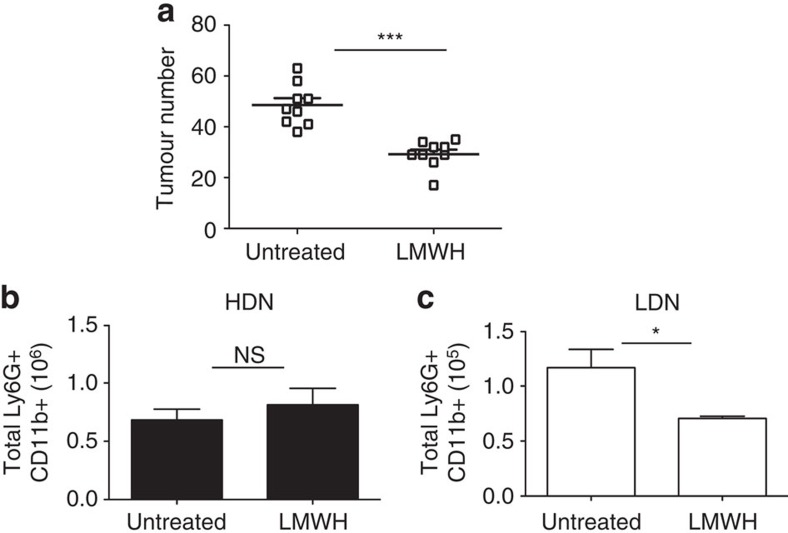
Treatment with LMWH reduces intestinal tumour burden and accumulation of protumorigenic LDNs in APC^Min/+^ mice. (**a**) Seven-week-old APC^Min/+^ mice were treated for 12 weeks with vehicle or with 90 IU per week LMWH via osmotic pumps. At the end of the treatment, mice were killed and intestinal tumours counted. (**b**,**c**) Blood was collected by heart puncture from mice treated as in **a**, separated on a Ficoll gradient and numbers of total HDNs and LDNs were determined by flow cytometry. Results are pooled from two independent experiments (*n*=9 mice per group). Significance was calculated by using unpaired *T*-test (NS, not significant; *P<0.05 and ***P<0.001). In panel **a** horizontal lines indicate mean with standard deviation (s.d.). Panels **b** and **c** bar graph show mean plus s.e.m.

As shown in Fig. 5a, APC^Min/+^ mice treated with LMWHs showed significantly fewer intestinal polyps as compared with animals treated with vehicle only. By contrast, warfarin did not lead to any effect on tumorigenesis (Supplementary Fig. 7).

Next, we assessed the number of LDNs and HDNs in LMWH-treated and vehicle-treated mice. Surprisingly, although we found no differences in the HDNs, we observed a significant decrease of LDNs in LMWH-treated mice (Fig. 5b,c). These results indicate that the reduction in tumour load in APC^Min/+^ mice treated with LMWHs was not due to an impaired neutrophil migration and confirm that increased coagulation and blood clots are responsible for the increase of LDNs and neutrophil dysfunction in tumour-bearing APC^Min/+^ mice. Further, not all anticoagulants are effective in reducing tumorigenesis.

### APC^Min/+^ mice undergo systemic complement activation

**Figure 6:**
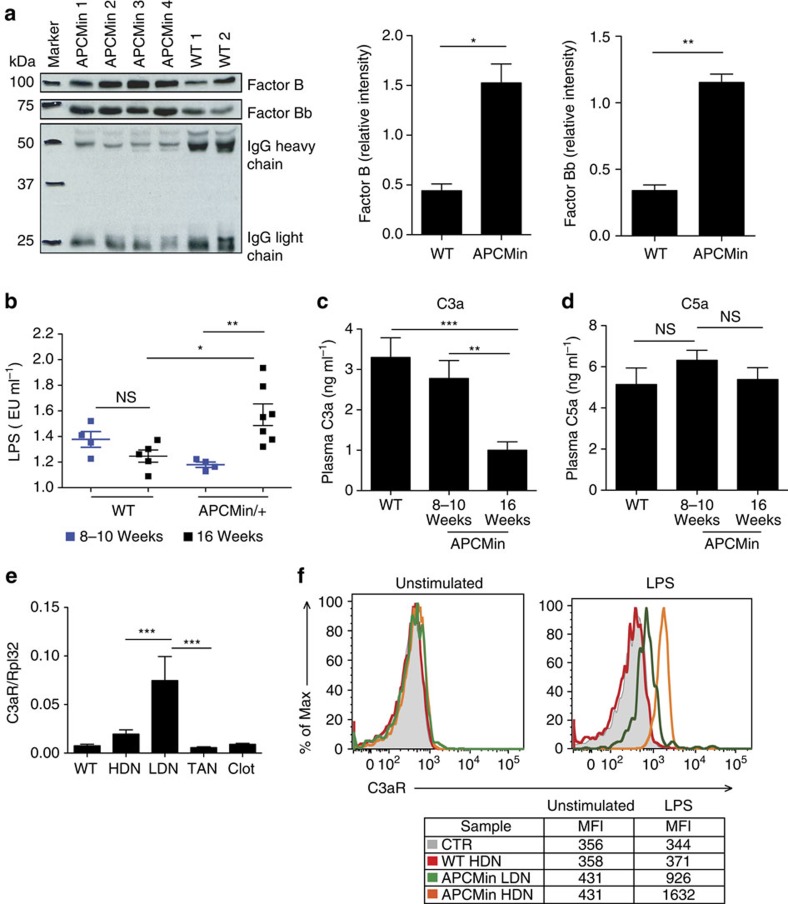
APC^Min/+^ mice undergo systemic complement activation via the alternative pathway. (**a**) Plasma was collected from 16-week-old APC^Min/+^ and WT mice, and assessed for complement activation by measuring Factor B (93 kDa) and Factor Bb (65 kDa) by western blotting. Immunoblottings were quantified by using ImageJ software and the results are represented as relative pixel intensity by using mouse IgG as internal standard. *N*=4–5 mice per group. (**b**) Endotoxin levels were measured in the plasma from young (8–10 weeks) and aged (16 weeks) APC^Min/+^ and WT mice by using the limulus amoebocyte lysate (LAL) test. *N*=4–7 mice per group. (**c**,**d**) C3a and C5a levels were quantified by ELISA assay in the plasma of 8- to 10-week-old and 16-week-old APC^Min/+^ mice and WT littermates. (**e**) C3aR levels were measured by real-time PCR in HDNs, LDNs, TANs and clot-treated BM neutrophils in WT and in APC^Min/+^ mice. (**f**) C3aR expression at the protein level was evaluated in HDNs from WT and in HDNs and LDNs of APC^Min/+^ mice by flow cytometry before (unstimulated) or after 1 h stimulation with 5 μg ml^−1^ LPS. Histograms show the mean fluorescence. HDNs from C3aRko mice were used as negative control. *N*=3–4 mice per group. Results are representative of two independent experiments. Significance was calculated by using two-way analysis of variance with Bonferroni post test (NS, not significant; *P<0.05, **P<0.01 and ***P<0.001). Bar graphs summarize mean plus s.e.m.

Complement can contribute to thrombosis both indirectly by increasing inflammation and directly by enhancing thrombus formation^37^. It has been shown that the anaphylatoxin C3a, one product of C3 cleavage, is able to enhance platelet aggregation, leading to thrombosis^38^. LMWHs can inhibit complement activation, an effect that is not shared by other anticoagulants^39^. Our finding that LMWH but not warfarin could affect tumour number suggested the involvement of complement activation in tumorigenesis. Interestingly, we found higher levels of complement Factor B and its activation fragment, factor Bb, in the plasma of APC^Min/+^ mice as compared with age-matched WT littermates (Fig. 6a and Supplementary Fig. 8). This finding confirms systemic complement activation via the alternative pathway. In addition, given the gut permeability defects of APC^Min/+^ mice^40^, we hypothesized that there could be an increase in circulating lipopolysaccharide (LPS) responsible for the activation of the complement cascade via the alternative pathway. Therefore, we analysed LPS plasma levels at the initial stage of tumorigenesis (8–10 weeks) and at its peak (16 weeks). We found in 16-week-old APC^Min/+^ mice a significant increase in serum levels of LPS as compared with that in WT mice (Fig. 6b). Subsequently, we evaluated the levels of complement anaphylatoxin C3a and C5a in the plasma of APC^Min/+^ mice and their WT littermates. We found a striking reduction of C3a in tumour-bearing 16-week-old APC^Min/+^ mice (Fig. 6c,d). This phenomenon might suggest an increased consumption of C3a in 16-week-old APC^Min/+^ mice through engagement with its receptor as already described for C5a^41^. Data in literature indicate that the C3aR is expressed on macrophages, platelets and mast cells, whereas its expression on granulocytes is still controversial^42^. At the mRNA level, we found the highest expression of *c3ar* but not *c5ar* on LDNs (Fig. 6e and Supplementary Fig. 9a). When we assessed C3aR expression at the protein level by using a monoclonal antibody by flow cytometry, we found that in unstimulated conditions neutrophils had undetectable levels of C3aR as previously reported^43^. However, after stimulation with LPS, HDNs and LDNs isolated only from APC^Min/+^ mice upregulated C3aR, with the HDNs being the population expressing the highest levels of the protein (Fig. 6f). The absence of C3aR on WT neutrophils despite the very low levels of mRNA does not rule out the expression of the protein at levels that are undetectable by flow cytometry. Altogether, these results demonstrate that LPS might be involved in the initiation of the alternative complement pathway and in the upregulation of C3aR on neutrophils in APC^Min/+^ mice.

### Absence of C3aR reduces tumours and neutrophils in APC^Min/+^

**Figure 7:**
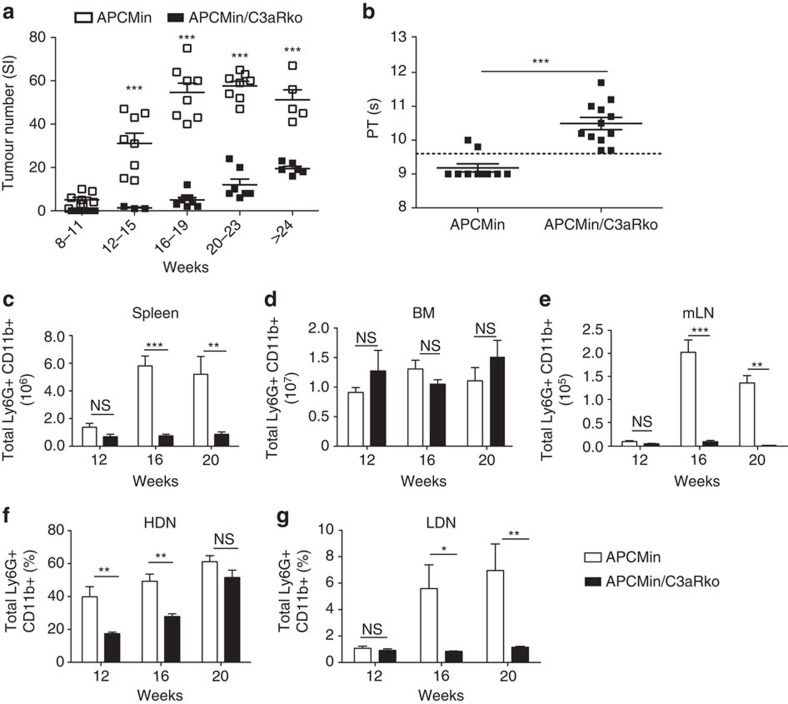
Knockout of the C3aR in APC^Min/+^ mice reduces tumour burden and protumorigenic neutrophils in peripheral blood and lymphoid organs. (**a**) APC^Min/+^ and APC^Min/+^/C3aR^−/−^ mice were killed at the indicated ages and tumours in the small intestine were counted. Data are representative of eight mice per group. (**b**) Coagulation measured by PT in 12-week-old APC^Min/+^ and APC^Min/+^/C3aR^−/−^ mice by using a coagulometer. Data are representative of three independent experiments. Significance was calculated by using unpaired *T*-test (***P<0.001). (**c**–**g**) Single-cell suspensions were prepared from the spleen, BM, mLN and peripheral blood of APC^Min/+^ and APC^Min/+^/C3aR^−/−^ mice at the indicated ages, and neutrophils assessed by flow cytometry. Bar graphs show mean number plus s.e.m. in organs or frequencies in blood of CD45.2+ Ly6G+ CD11b+ neutrophils. A minimum of six mice per group was used and results are pooled from two independent experiments. Significance was calculated by using two-way analysis of variance (ANOVA) with Bonferroni post test (NS, not significant; *P<0.05, **P<0.01 and ***P<0.001).

To assess the role of C3a-C3aR axis during intestinal tumorigenesis, we genetically disrupted C3aR signalling in APC^Min/+^ mice. This genetic manipulation did not result in any weight change in the mice (data not shown). APC^Min/+^/C3aR^−/−^ and APC^Min/+^ littermates were killed at different ages and polyps in the small intestine were examined. As shown in Fig. 7a, APC^Min/+^/C3aR^−/−^ developed significantly fewer polyps as compared with the APC^Min/+^ mice. Next, we tested whether the reduced tumorigenesis was a consequence of reduced neutrophil recruitment into the small intestine. In a model of *Salmonella*-induced enteritis, we found no difference between WT and C3aR^−/−^ mice in the number of neutrophils in the lamina propria of the small intestine (Supplementary Fig. 9b). Therefore, the absence of a functional C3a signalling does not impair the migration of neutrophils to the inflammatory site.

In addition to the reduced tumour burden, we noticed that APC^Min/+^/C3aR^−/−^ were mostly healthy and did not show anaemia, while APC^Min/+^ mice developed severe cachexia and anaemia around 20 weeks of age^23^. Most interestingly, aged APC^Min/+^/C3aR^−/−^ showed normalized PT (Fig. 7b). We further investigated the occurrence of neutrophilia in APC^Min/+^/C3aR^−/−^ as compared with that in APC^Min/+^. As shown in Fig. 7c–e, no increase in neutrophil numbers was found in the spleen, BM and mLN of APC^Min/+^/C3aR^−/−^ as compared with their APC^Min/+^ littermates. Finally, the amount of LDNs in APC^Min/+^/C3aR^−/−^ was not elevated at all time points (Fig. 7f,g), whereas HDNs were increased only in 20-week-old mice.

These findings strongly suggest that complement activation and C3a release induce hypercoagulation and subsequent thrombus formation in APC^Min/+^ mice that skews neutrophils towards an N2 LDN phenotype and ultimately favours tumour progression.

### LDNs are prone to produce NETs via a C3aR-dependent mechanism

**Figure 8:**
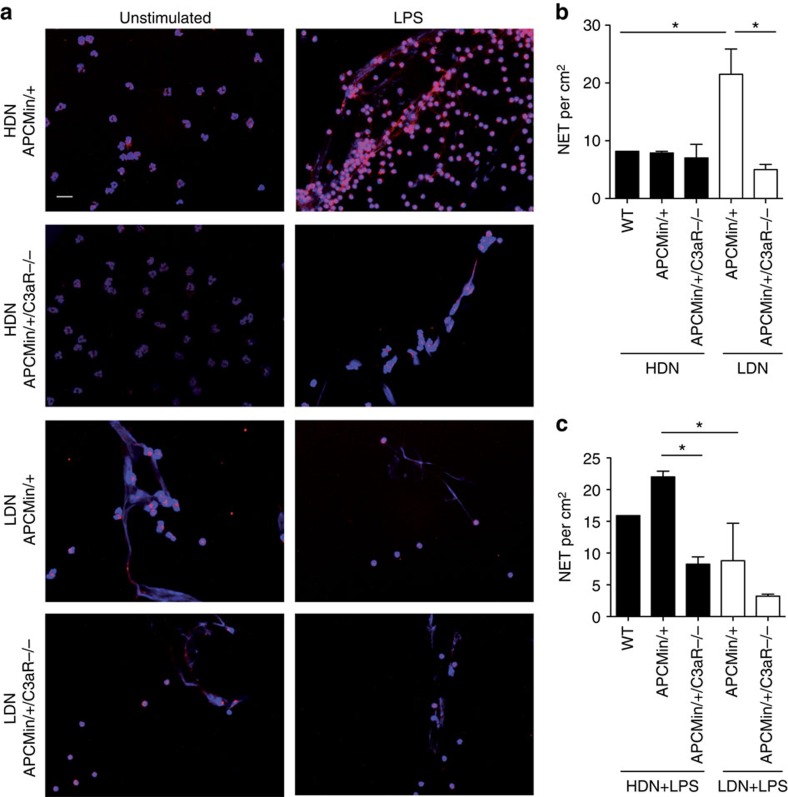
LDNs are prone to produce NETs via a C3aR-dependent mechanism. (**a**–**c**) Unstimulated HDNs and LDNs purified from 16-week-old APC^Min/+^, APC^Min/+^/C3aR−/− and WT mice (only HDNs) were stained with DAPI (4,6-diamidino-2-phenylindole; blue) and MPO (red), and visualized by widefield fluorescence microscopy. In **a**, representative merged pictures are shown for HDNs and LDNs of APC^Min/+^ and APC^Min/+^/C3aR−/− mice. Original magnification, × 20. Scale bar, 40 μm. (**b**–**c**) For quantification, NETs were counted on the whole slide and expressed as NET per cm^2^. Results are pooled from two independent experiments with two to three mice per group. Significance was calculated by using one-way analysis of variance (ANOVA) with Bonferroni post test (*P<0.05). Data in panels b and c is summarized as mean plus s.e.m..

Neutrophils can undergo NETosis, a process involved in trapping extracellular microorganisms^44^, but also implicated in thrombus formation and the coagulation process^45^. NET formation has been described also in several tumour models^46^,^47^. Having shown that in the absence of C3aR mice display reduced LDNs, develop fewer tumours and are less susceptible to hypercoagulation, we wondered whether signalling via C3aR may be involved in neutrophil activation and NET formation. Neutrophils (HDNs or LDNs) from APC^Min/+^ mice, APC^Min/+^/C3aR^−/−^ or WT were assessed for their capacity to form NETs. As shown in Fig. 8a–c, we found that LDN from APC^Min/+^ mice underwent unprompted NETosis. This observation mirrors a finding described in several autoimmune diseases such as psoriasis and systemic lupus erythematosus, where LDNs spontaneously undergo NETosis^48^. Further, the ability of LDNs was lost in the absence of C3aR signalling. By contrast, HDNs underwent NETosis only after LPS stimulation and the absence of C3aR abrogated this capacity (Supplementary Fig. 10).

These results suggest that the NETosis resulting from the stimulation of HDNs with LPS might be responsible for the hypercoagulation in APC^Min/+^ mice. In contrast, in the absence of C3aR, the ability of HDNs to respond to LPS is reduced, NET production is impaired and also the number of LDNs remain low as a consequence of reduced thrombi formation. Altogether, these observations may explain why APC^Min/+^/C3aR^−/−^ mice have reduced coagulation defects and tumour burden.

### Small bowel cancer show neutrophilia and hypercoagulation

Having observed a clear correlation between neutrophilia and hypercoagulation in the APC^Min/+^ mouse model, we wondered whether a similar link was observed in human intestinal tumorigenesis. To this aim, we analysed retrospectively over a time frame of 15 years 478 patients with intestinal tumours, of which 466 patients were diagnosed with CRC and 12 patients were diagnosed with small bowel cancer (Supplementary Table 1). The difference in cohort size mirrors the rarity of small intestinal cancer over CRC. Very interestingly, we found that at the time of diagnosis, 6 out of 12 patients with small intestinal cancer displayed blood neutrophilia and hypercoagulation that could not be normalized even after oral anticoagulant treatment (warfarin) (Table 1). Notably, during the follow-up 83% (10 out of 12) of these patients underwent hypercoagulation and neutrophilia with consequent increase of the neutrophil/lymphocytes ratio (NLR).

In contrast, in CRC, we could not detect any hypercoagulation and neutrophilia, independently on the disease stage. By adjusting for confounders and other prognostic factors, we found that NLR was the only factor associated with pathologic tumour stage (Supplementary Table 2). In particular, an NLR >5 was associated with an almost threefold increase in pathologic tumour (Supplementary Table 3). However, it is important to note that the increased NLR in CRC patients was primarily due to a reduced lymphocyte rather than an increased neutrophil frequency. Altogether, these results suggest that hypercoagulation and neutrophilia are clinical traits of small bowel cancer and do not correlate with the disease stage in CRC. This is consistent with the recent finding that only polymorphisms in factor V Leiden and not other clotting factors showed an increased risk (5.8-fold) for CRC compared with non-carriers^49^.

## Discussion

Cancer patients often develop haemostatic defects that can be exacerbated by chemotherapy and ultimately lead to VTE complications, which are the second most common cause of cancer-related death^1^. This is supported by clinical evidence, demonstrating that tumour progression and metastasis formation is facilitated by cancer-associated hypercoagulation^50^. Until recently, it was unclear whether the persistent activation of the coagulation pathway caused or was a consequence of tumour development^51^. Initial clues that coagulation may be linked to tumorigenesis comes from studies overexpressing the human oncogene MET in somatic cells of the liver, which resulted in hypercoagulation and internal haemorrhage, and correlated with tumour development^5^.

Our work demonstrates that during intestinal tumorigenesis, hypercoagulation can directly affect neutrophil effector function and is linked to complement activation. We found that APC^Min/+^ mice undergoing spontaneous intestinal tumorigenesis show increased activated peripheral neutrophils (LDNs) and TANs. LDNs with immunosuppressive functions have been recently described in patients following septic shock, a medical condition associated with hypercoagulation^52^,^53^. Furthermore, immunosuppressive LDNs that impair T-cell proliferation through local depletion of L-arginine have been described in several cancer patients^54^,^55^,^56^. LDNs differ from granulocytic myeloid-derived suppressor cells, as they have a reduced ability in ROS production, similar to TANs^12^. We were able to reproduce the N2 phenotype *in vitro* by treating naive BM neutrophils from APC^Min/+^ and WT animals with blood clots. Clot-treated neutrophils displayed upregulation of arginase-1, whose enzymatic activity has been shown to be induced by thrombin in endothelial cells^55^,^57^.

We also demonstrated that the concomitant development of neutrophilia and hypercoagulation are specific for cancer of the small intestine but not colon cancer. This finding is in line with a recent population-based study in which the incidence of coagulation defects and subsequent VTE in CRC patients significantly decreased six months after diagnosis, despite cancer progression^58^. Although its incidence has been rising in recent years, small intestinal cancer represents only 2% of cancers of the digestive system and the underlying molecular mechanisms are still poorly understood^59^. Owing to the absence of effective screening and the nonspecific symptoms of this type of cancer, these tumours are detected at an advanced stage. However, even at late stages, CRC is not characterized by hypercoagulation or neutrophilia, thus suggesting that these two pathologies are aetiologically distinct.

We found that treatment of APC^Min/+^ mice with LMWH could drastically reduce tumours and LDN numbers with no effect on HDNs. LMWH has been described to inhibit both complement activation^39^ and inflammatory responses^60^. The effect on inflammatory responses could also be linked to the inhibition of complement activation, as mice lacking C3 show reduced production of the inflammatory cytokine interleukin-1β by neutrophils^61^. However, this process appears to be dependent on C5a production, as it is phenocopied in C5aR^−/−^ mice, which also display reduced colitis-associated carcinogenesis^61^.

Complement can significantly contribute to thrombosis by enhancing blood clotting. For instance, the anaphylatoxin C3a, the end product of C3 activation, can lead to thrombus formation^62^ and, conversely, coagulation factors such as thrombin, human coagulation factors (F) XIa, Xa and IXa, and plasmin can effectively cleave C3 and C5 (refs 37, 63). In addition, C3a-like fragments have been found in blood clots, further confirming local complement activation and deposition^63^. In agreement with these observations, we found that C3a was required for tumorigenesis and development of coagulation defects. Wu *et al.*^64^ recently described a protective effect of the C3a/C3aR axis in an acute model of intestinal ischaemia-reperfusion injury, which was mainly due to the induction of an inflammatory response. In our model, tumorigenesis does not rely on overt inflammation but on coagulation and the consequent induction of a N2 phenotype in neutrophils.

**Figure 9:**
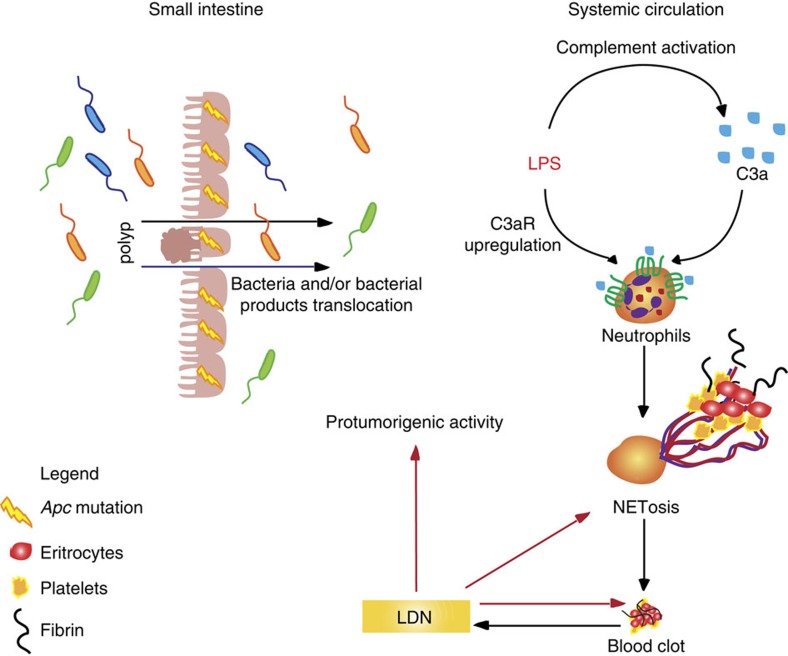
Neutrophils and coagulation during small intestinal tumorigenesis. The Apc mutation induces growth of polyps in the small intestine of APC^Min/+^ mice and results in defects in gut permeability, ultimately leading to bacterial and/or bacterial products translocation in the systemic circulation. LPS induces upregulation of C3aR on neutrophils (HDNs and LDNs), thereby enhancing the interaction with C3a produced via alternative complement activation. These events result in NET induction, which form a scaffold for the recruitment of components of the coagulation cascade. By interacting with the circulating neutrophils, the formed clot induces LDNs, which can further fuel coagulation in a positive feedback loop by undergoing NETosis and can exert protumorigenic functions.

We observed increased levels of LPS in the plasma of APC^Min/+^ mice experiencing hypercoagulation. Low doses of LPS have been shown to predispose neutrophils in tumour-bearing mice to NET formation, which can also contribute to cancer-related thrombosis^18^,^65^. *In vivo* data demonstrated that during *Staphylococcus aureus* infection, NETosis requires C3, and that C3aR played a greater role as compared with C5aR in NET formation^66^. Our *ex vivo* observation that C3aR is induced by LPS suggests a regulatory feedback loop between neutrophils and complement activation (Fig. 9). In this feedback circle, neutrophils become more susceptible to C3a activation following LPS stimulation and produce NETs. This results in thrombi formation, which are responsible for inducing a protumorigenic phenotype in the neutrophils, as seen in LDNs. In addition to their protumorigenic phenotype, LDNs undergo spontaneous NETosis, further exacerbating hypercoagulation.

Thrombus formation and hypercoagulation would normally occur during infection, to trap the microorganisms in the NETs. We propose that the immune system to shut down this reaction induces the development of neutrophils with an N2 phenotype in response to the newly generated blood clots. In a mutation-dependent protumorigenic environment, these N2 neutrophils would fuel tumour growth. Interventions aimed at blocking complement activation such as inhibition of C3aR signalling or LMWH administration can affect coagulation, NETosis and LDN formation, thus having an impact on tumour growth. TF has been shown to participate in complement-induced coagulation^67^ and, indeed, APC^Min/+^ mice treated with recombinant nematode anticoagulant protein c2, an inhibitor of TF, showed reduced tumorigenesis^68^.

Our findings provide a novel mechanistic link between the pro-tumorigenic effects of hypercoagulation and neutrophil function in cancer, and highlight the potential use of LMWH not only to prevent VTE but also to contrast the hypercoagulation induced by the complement–neutrophil axis. Considering the limited availability of effective tools to diagnose small intestinal tumours, we propose that hypercoagulation, neutrophilia and the presence of LDNs in the blood may be used as a new biomarker for the early diagnosis of small intestinal tumours.

## Methods

### Animals

*C57BL/6J-ApcMin/J* (referred to as APC^Min/+^) and *C57BL/6J* (referred to as WT) mice were bred and maintained in our SPF (Specific Pathogen Free) animal facility. C3aR−/− mice on C57BL/6J background were a kind gift from Dr Bao Lu. *APC*^*Min/+*^*/C3aR−/−* mice were obtained by crossing *C57BL/6J-ApcMin/J* with *C3aR−/−* mice in our animal facility. Experiments were performed by using male mice, unless otherwise specified. All animal experiments were performed in accordance with the guidelines established in the Principles of Laboratory Animal Care (directive 86/609/EEC). Specifically, the project was notified to the Italian Ministry of Health before the implementation of the current legislation in accordance with the Directive 2016/63/EU of the European Parliament. Since the new law does not apply to projects approved before its enactment, for the animal experiments shown in this manuscript a notification to the Italian Ministry of Health was the only requirement.

### Patients

The patient data presented in this study were obtained by using the cancer registry of the European Institute of Oncology. The small intestinal cancer cohort included 12 patients who were diagnosed between 1999 and 2014. The CRC cohort included 466 patients recruited during the same period. Registry information included basic demographic such as sex and age, date of diagnosis and cancer histology. The date of cancer diagnosis was defined as the earliest date reported in the registry or the date of hospital admission. Accordingly, the reported International Normalized Ratio, neutrophil values and NLRs were relative to the earliest date available. Patients diagnosed with papilla of Vater carcinoma were not included in this study. An informed consent was signed by participants and the use of the patient data included in the IEO Tumour Registry was approved by the IEO Institutional Review Board (March 2013).

### Flow cytometry and cell sorting

To prepare single-cell suspensions from mouse organs, the spleen and mLNs were mashed through a 40-μm cell strainer by using a syring plunger. For the BM, after removing muscle and connective tissue, both ends of the tibia and femur were cut and cells were flushed out by using a 27-gauge needle attached to a syringe containing complete medium. For isolation of lamina propria and tumour-associated immune cells, intestine or polyps were shaken in PBS, 1% BSA and 10 mM EDTA, to remove epithelial cells, and further digested for 30 min at 37 °C with Collagenase VIII (Sigma-Aldrich) in complete medium with shaking. The harvested cells were subsequently submitted to a discontinuous Percoll gradient before sorting of neutrophils. For isolation of cells from peripheral blood, eritrocytes were lysed with RBC buffer (Sigma-Aldrich). Alternatively, peripheral blood cells were separated by Ficoll gradient centrifugation and both high-density (fraction co-purifying with the erytrocytes) and low-density (fraction co-purifying with mononuclear cells) fractions were collected for further analysis and neutrophil enrichment.

For flow cytometry staining or cell sorting, cells were incubated with anti-FcR antibody (clone 24G2) and stained with the following primary antibodies: anti-CD45.2 (1:300; clone 104, eBioscience), CD45.1 (1:300; clone A20), Ly6G (1:200; clone 1A8), CD3 (1:300; clone 17A2, eBioscience), Ly6C (1:200; clone AL-21), CD11b (1:200; clone M1/70, eBioscience), CD4 (1:300; cloneRM4-5), CD8a (1:200; 53-6-7), CD49b, NK1.1 (1:100; clone PK136), CD19 (1:200; clone 1D3), B220 (1:200; clone RA3-6B2), I-A/I-E (1:200; clone M5/114.15.2) and CD11c (1:100; clone HL3). For C3aR staining, peripheral blood leukocytes were labelled with a rat monoclonal anti-C3aR antibody (1:100; clone 14D4, Hycult) followed by a goat anti-rat secondary antibody (1:800; Invitrogen). All antibodies were purchased from BD Pharmingen, unless otherwise specified. Samples were acquired with FACSCanto II or FACSCalibur (BD Pharmingen) and analysed with FlowJo software (TreeStar).

Cells were sorted by FACSVantage (BD Biosciences) into CD45.2^+^CD3^−^Ly6G^+^CD11b^+^ and collected in complete medium (RPMI, 5% fetal bovine serum, 1% penicillin–streptomycin and 1% glutamine. Purity was>97%.

### RNA extraction and real-time PCR

RNA from sorted or Microbeads-purified neutrophils was extracted by using RNeasy Plus Mini Kit or, for <500,000 cells, with RNeasy Plus Micro Kit (Qiagen). RNA concentration was measured by Nanodrop. RNA integrity was assessed by Agilent 2,100 Bioanalyzer (Agilent Technologies) and only RNA with RNA Intergrity Number (RIN) ≥8 were used for further analysis. Reverse transcription for complementary DNA synthesis was performed with ImProm Reverse transcription kit (Promega) by using random primers. For very low amount of RNA (<10 ng ml^−1^), the cDNA was synthesized by using High-Capacity cDNA Reverse Transcription kit (Applied Biosystem). Real-time PCR was performed on cDNA using SYBR green chemistry (Applied Biosystems) and commercially available Quantitect primers (Qiagen) or previously published primer sets from PrimmBiotech (specific sequences are reported in the ‘Primer sequences' section). Reactions were run on a real-time PCR system (ABI7500; Applied Biosystems). Samples were normalized to *rpl32*.

### Coagulation and blood parameters

PT was assessed in WT and tumour-bearing mice by using the CoaguCheck System XS (Roche) through the application of a drop of non-anticoagulated blood on test strips.

HGB, mean platelet volume, RBCs and platelet count were assessed by Cell-Dyn Sapphire (Abbott Diagnostics Division, Santa Clara, CA). HGB was measured after a dilution procedure with repetitive absorption spectrometry at a wavelength of 540 nm. Fibrinogen was measured by using the ACL TOP 500 (Instrumentation Laboratory, Bedford, MA, USA), a fully automated random-access multiparameter coagulation analyser, equipped with a photo-optical clot-detection unit. For HGB, mean platelet volume, RBCs and platelet count, EDTA was used as anticoagulant. For fibrinogen measurements, the blood was mixed at 1:9 ratio (anticoagulant:blood) with sodium citrate.

### BM chimeras

Untouched Lin− cells were obtained from the BM of 7-week-old APC^Min/+^ and WT (CD45.1) mice by using the Lineage Cell Depletion kit (Miltenyi). Lin− BM cells (5 × 10^5^) were transplanted into the lateral tail vein of lethally irradiated (10 Gy) APC^Min/+^ and CD45.1 congenic recipient mice. Blood chimerism was checked after 4 weeks. Chimeric animals were killed 13 weeks after transplantation, for analysis of neutrophils and tumour count.

### Neutrophil isolation and *in vitro* culture

Neutrophils were isolated from the BM, peripheral blood and polyps. For stimulation with blood clots, neutrophils were purified from the BM by using Ly6G microbeads (Miltenyi), unless otherwise specified, and cultured in complete medium for 12 h. Subsequently, neutrophils were harvested and lysed in RLT buffer for RNA extraction. Blood clots were obtained from non-anticoagulated blood left at 37 °C, to allow coagulation, and were washed in PBS before co-culture with neutrophils. Equal amounts of anticoagulant-treated blood (unclotted blood) was used as control in quantitative PCR and ROS production experiments. For transwell experiments, 5 × 10^5^ purified neutrophils were seeded in the bottom well and blood clots were placed in the insert containing a cell-impermeable membrane (0.4 μm). Cells were cultured for 12 h. To isolate RNA from blood clot-derived cells after culture with neutrophils, we prepared blood clots from CD45.2-positive APC^Min/+^ mice and purified CD45.1-positive neutrophils from WT mice. After 12 h stimulation, the cells were stained with anti-CD45.1 fluorescein isothiocyanate-conjugated (1:300; BD Pharmingen) and subsequently magnetically separated by using anti-fluorescein isothiocyanate microbeads (Miltenyi). CD45.1 neutrophils and CD45.2 clot-derived cells were then used for RNA extraction.

For evaluation of ROS production, FACS-sorted TANs and neutrophils purified from peripheral blood were stimulated for 20 min with 30 ng ml^−1^ (unless otherwise specified) of PMA in PBS and 5% fetal bovine serum. Subsequently, they were pulsed with 1 μM of dihydrorhodamine (Invitrogen) for 15 min before evaluation of ROS production by FACSCalbur or FACSCanto II (BD Bioscience).

### TGF-β1 in blood clots

Blood clots from APC^Min/+^ and WT littermates were prepared as described in the previous paragraph, weighed, seeded in 96-well U-bottom plates in RPMI and 5% FCS, and cultured overnight at 37 °C. Subsequently, plates were briefly centrifuged at 300 *g* and supernatants were collected for TGF-β1 assessment by using DuoSet mouse TGF-β1 ELISA kit (R&D) according to manufacturer's instructions.

### LMWH and warfarin treatments

For LMWH treatment, 7-week-old APC^Min/+^ mice were treated with 90 IU per week of Enoxaparin (Clexane, Sanofi-aventis) administered s.c. in mini osmotic pumps (Alzet) with a mean pumping rate of 0.16 μl h^−1^ over a period of 12 weeks. The osmotic pumps were loaded and implanted according to the manufacturer's instruction manual. Briefly, for the implantation of osmotic pumps, mice were anaesthetized with 2.5% Avertin and an incision was performed on their backs, to allow the correct positioning of the pump. The amount of LMWH released was verified at the end of the experiment by measuring the remaining volume in the pump reservoir.

For warfarin treatment, 7-week-old APC^Min/+^ mice were administered 2.5 mg l^−1^ of (S)-warfarin in their drinking water for 5 days followed by 2 days of water (Sigma-Aldrich) over a period of 12 weeks. Mice drinking normal water served as controls. At the end of the experiment, mice were killed and intestinal polyps counted.

### Complement activation

For analysis of systemic complement proteins, blood was collected by cardiac puncture in 25 mM EDTA plus 50 μg ml^−1^ Futhan (FUT-175, BD Bioscience), to avoid unwanted *ex-vivo* complement activation and immediately centrifuged at 4 °C. Plasma samples were aliquoted and stored at −20 °C until use.

Mouse complement C3a and C5a Elisa kits (USCN Business Co., Ltd) were used for plasma C3a and C5a quantification, according to manufacturer's instructions.

For western blotting, plasma proteins were quantified by Bradford assay (BioRad). Thirty micrograms of protein were separated by SDS–PAGE on a 10% polyacrilamide gel and transferred onto a nitrocellulose membrane. The membrane was blocked in 5% milk at room temperature for 1 h, probed overnight at 4 °C with primary antibody rabbit anti-mouse Factor B/Bb (1:500, Cederlane) and washed extensively before incubating it with anti-rabbit horseradish peroxidase (1:15,000, Cell Signaling). Proteins were visualized by using ECL detection reagent (GE Healthcare, Amersham). Uncropped pictures of the western blotting are provided in the Supplementary Figures. Precision Plus Protein Dual Color Standard (BioRad) was used as molecular weight marker. Immunoblottings were quantified by using ImageJ software and results are represented as relative pixel intensity by using mouse IgG as the internal standard.

### Plasma endotoxin levels

Plasma endotoxin content was determined by using the limulus amoebocyte lysate assay (Lonza). Briefly, defrosted plasma was diluted 1:10 in pyrogen-free water and then heated at 70 °C for 10 min, to inactivate potential endotoxin-neutralizing agents. Fifty microlitres of heat-inactivated plasma was combined with 50 μl limulus amoebocyte lysate reagent for 10 min at 37 °C and 100 μl of substrate solution were added before reading the absorbance at 405 nm. Plasma endotoxin concentrations were estimated by using a standard curve prepared from kit-supplied *Escherichia coli* reference standard endotoxin in the same plate.

### Visualization and quantification of NETs

To evaluate NET formation, purified HDNs and LDNs were seeded onto poly- L-lysine-coated chamber slides and left unstimulated or stimulated for 3 h with 10 μg ml^−1^ LPS in RPMI 1% FCS. After incubation, neutrophils were fixed in 3% paraformaldehyde, permeabilized and incubated overnight with anti-myeloperoxidase antibody (1:100; MPO, rabbit polyclonal, Abcam). A Cy3-conjugated donkey anti-rabbit IgG was used as secondary antibody (dilution 1:400). Slides were counterstained with 4,6-diamidino-2-phenylindole and mounted with Vectashield (Vector Laboratories, Inc.). NETotic areas characterized by co-staining of extracellular DNA and MPO were visualized by using a widefield fluorescence microscope (Olympus BX61) with MetaMorph software.

For quantification, NETs were counted on the whole slide and expressed as NET per cm^2^.

Images were analysed by using ImageJ software, and the same threshold and contrast setting were applied to each image within a given experiment.

### Immunohistochemistry

Intestinal tumours were fixed in Hollandes fixative (Polysciences, Inc.) and subsequently embedded in paraffin. For immunohistochemistry staining, sections were deparaffinized, rehydrated and treated with 3% H_2_O_2_ before performing specific staining with rat anti-mouse Ly6G (1:200) followed by the secondary antibody, anti-rat IgG horseradish peroxidase-conjugated (1:1,000; R&D). Visualization was obtained with DAB substrate (Vector Laboratories). After counterstaining with haematoxylin, sections were dehydrated and prepared for visualization by mounting with mounting medium Eukitt (Sigma-Aldrich).

### BrDU assay

Twelve- and 16-week-old APC^Min/+^ mice and WT littermates were intraperitoneally injected with 1.5 mg of BrDU (Sigma-Aldrich). Mice were killed 24 h later, and the blood, spleen and BM were collected to prepare single-cell suspensions. BrDU incorporation was evaluated in CD3^−^ CD11b^+^Ly6G^+^ neutrophils by FACS analysis with the BrDU flow kit (BD Pharmingen) according to manufacturer's instructions.

### Neutrophil mobilization by thioglycollate

Twelve-week-old WT mice were s.c. treated for 3 days with 30 IU of heparin or 30 IU of LMWH or PBS. Subsequently, mice were intraperitoneally injected with 3% thioglycollate and after 3 h peritoneal wash was performed by using PBS and 3 mM EDTA. Cell suspensions were washed, counted and stained with anti-CD3, CD11b and Ly6G.

### *Salmonella* infection

Twelve-week-old C3aRko mice and WT littermates were treated via oral gavage with 2 mg streptomycin and fed 500,000 CFU of *Salmonella* (SL1344) 20 h later. Twenty-four hours after infection, mice were killed and small intestines harvested, to prepare single-cell suspensions as previously described. Total numbers of CD3- Ly6G+ CD11b+ cells were evaluated by FACS analysis.

### Primer sequences

Primers from PRIMM (sequences 5′–3′) are as follows: *arg-1:* FW: AACACTCCCCTGACAACCAG, RV: GTTCCCCAGGGTCTACGTCT; *c5aR:* FW: GGTATTAACTATGGTGGGGGTAGC, RV: GCAGCCAGAAGATAAAGAAACAGA; *ccl2*: FW: CAGGTCCCTGTCATGCTTCT, RV: GTCAGCACAGACCTCTCTCT; *ccl5*: FW: ATATGGCTCGGACACCACTC, RV: CCACTTCTTCTCTGGGTTGG; *g-csf*: FW: GTCTCCTGCAGGCTCTATCG, RV: CTGGAAGGCAGAAGTGAAGG; *icam-1*: FW: TTCACACTGAATGCCAGCTC, RV: GTCTGCTGAGACCCCTCTTG; *inos*: FW: TTCCAGAATCCCTGGACAAG, RV: TGGTCAAACTCTTGGGGTTC; and *rpl32*: FW: AAGCGAAACTGGCGGAAAC, RV: TAACCGATGTTGGGCATCAG; *tnf-α* FW:TCTTCTCATTCCTGCTTGTGG, RV: CACTTGGTGGTTTGCTACGA.

Primers from Qiagen (Quantitect primer assay) are as follows: *c3ar1*: QT00251216; *mmp8*: QT00113540; *mmp9*: QT00108815; and *il10*: QT00106169.

### Statistics

Statistical significance between two groups was determined by the non-paired Student's *t*-test. The comparison of multiple groups was carried out by two-way analysis of variance followed by Bonferroni post test using GraphPad Prism software. *P<0.05, **P<0.01 and ***P<0.001; NS, not significant.

For the analysis of data in patients, results were represented as median and interquartile range. Coagulation factors were evaluated considering a median value per patient among the first four assessments in time. Cutoff point for NLR was chosen based on the literature^69^.

Risk analyses were conducted using logistic regression and odds ratios were calculated, to assess the probability of having higher degree of disease, adjusting for significant confounders (age, gender, BMI and sub-site) and other prognostic factors. Residual plots assessed the validity of model assumptions and transformations adopted to achieve normality. All statistical tests were two sided and *P*<0.05 was considered statistically significant. All data were found to fit a normal distribution. The statistical analyses were performed with the Statistical Analysis System Version 8.2 (SAS Institute, Cary, NC).

## Additional information

**How to cite this article:** Guglietta, S. *et al.* Coagulation induced by C3aR-dependent NETosis drives protumorigenic neutrophils during small intestinal tumorigenesis. *Nat. Commun.* 7:11037 doi: 10.1038/ncomms11037 (2016).

## Supplementary Material

Supplementary Information Supplementary Figures 1-10 Supplementary Tables 1-3.

## Figures and Tables

**Table 1:** Identification of coagulation defects and neutrophila in patients with small bowel cancer.

**Patient**	**Sex**	**Age (years)**	**Cancer localization**	INR (08–1.2) –(2–4)*	**NLR**	**NEU (40–74%)**
Pt 01	F	63	Ileum	1.1*	10.0	**84.5**
Pt 02	M	79	Duodenum	1.1	1.4	50.4
Pt 03	F	81	Ileum	1.0*	4.1	68.8
Pt 04	M	61	Duodenum	1.1*	6.9	**79.9**
Pt 05	M	64	Duodenum	1.1*	1.7	53.9
Pt 06	F	50	Ileum	1.0	5.2	**77.8**
Pt 07	M	57	Duodenum	1.3*	6.0	**81.5**
Pt 08	F	60	Duodenum	1.6*	11.0	**82.4**
Pt 09	M	69	Duodenum	1.1	1.6	52.5
Pt 10	F	44	Duodenum	1	2.6	63.8
Pt 11	M	76	Jejunum	1.1	2.2	56.9
Pt 12	F	54	Jejunum	0.9	1.6	55.1

F, female; INR, International Normalized Ratio; M, male; NEU, neutrophils; NLR, neutrophil/lymphocyte ratio.

The table shows the small bowel cancer patients included in this study. Measured INR for patients undergoing anticoagulant therapy is identified with an asterisk (*). The INR therapeutic range during anticoagulant therapy is 2–4. In the patients not receiving anticoagulants, the measured INR is contained in the normal INR range 0.8–1.2. The bold numbers indicate neutrophil percentages above the normal range.
