# Supplementary material for: Coagulation induced by C3aR-dependent NETosis drives protumorigenic neutrophils during small intestinal tumorigenesis
Source: Nat Commun. 2016 Mar 21;7:11037. doi: 10.1038/ncomms11037 (PMC4802169; doi:10.1038/ncomms11037)
Supplement: Supplementary Information — Supplementary Figures 1-10 Supplementary Tables 1-3. [file ncomms11037-s1.pdf]

SUPPLEMENTARY INFORMATIONS

Supplementary Figure 1

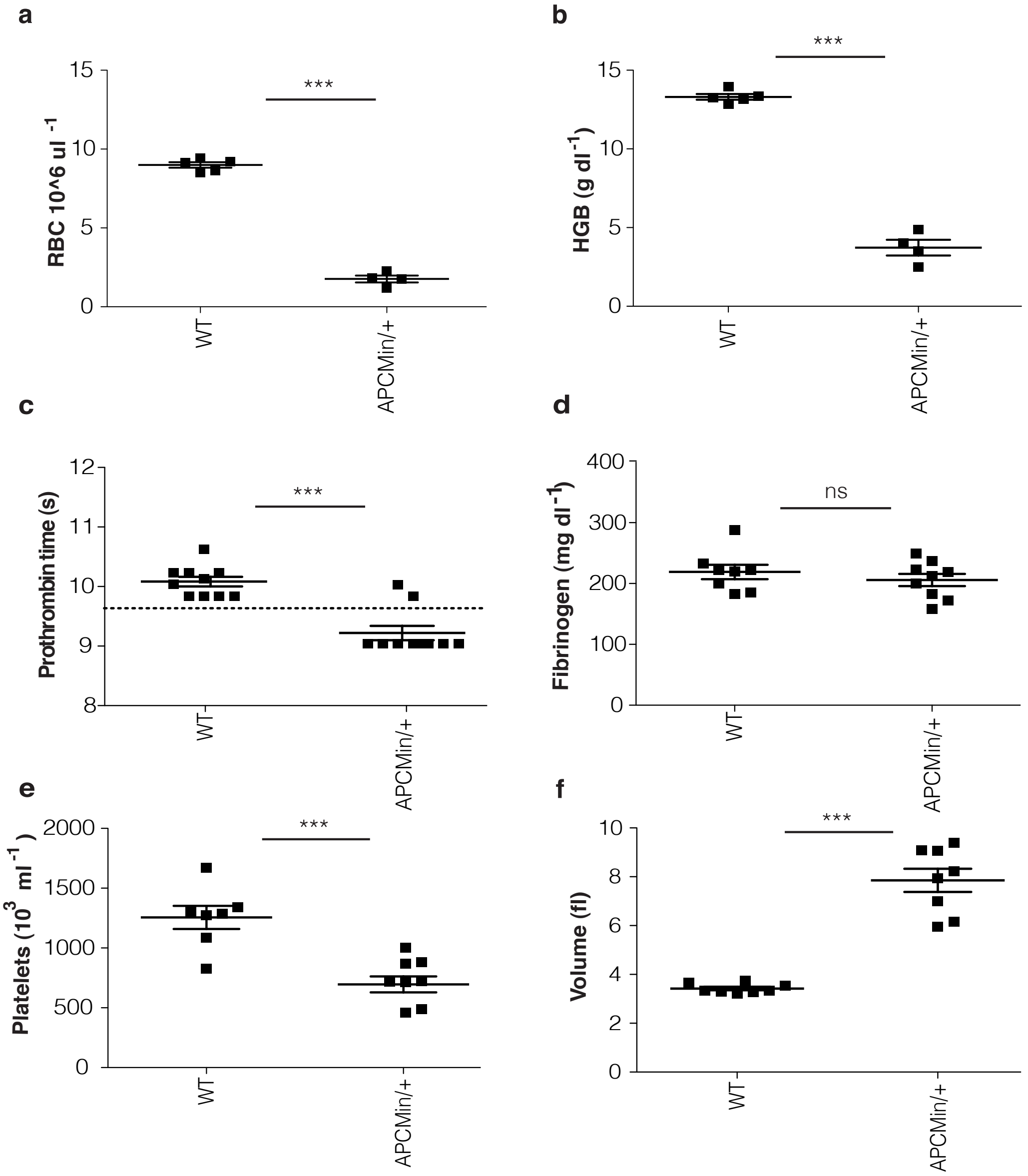

APC$^{Min/+}$ mice develop hypercoagulation

(a-d) APC$^{Min/+}$ mice and WT littermates were sacrificed between 12 and 16 weeks of age and blood was collected by heart puncture for assessment of (a) red blood cells and (b) haemoglobin. (c) Prothrombin times prior to sacrifice were determined by using a coagulometer. (d) After sacrifice fibrinogen levels, (e) platelet numbers and (f) platelet volume were measured. N=5-10 mice/group. Results are representative of two independent experiments. Significance was calculated by using unpaired T-test (ns= not significant; *** $p< 0.001$).

Supplementary Figure 2

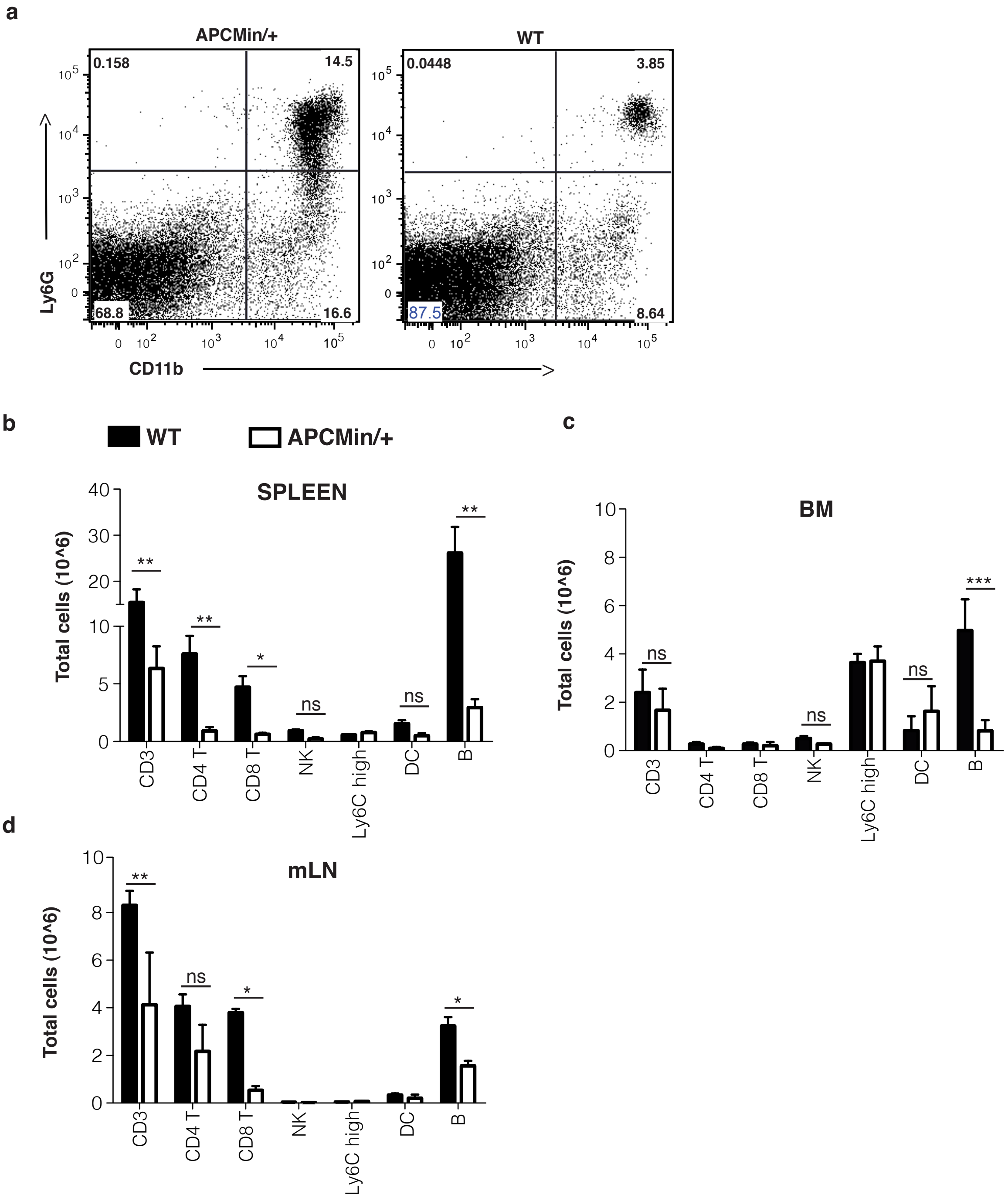

Aged $APC^{Min/+}$ show neutrophils accumulation and lymphodepletion in BM and secondary lymphoid organs.

$APC^{Min/+}$ mice and WT littermates were sacrificed at 16 weeks of age and single cell suspensions were obtained from spleen, BM and mLN. (a) Representative plots of neutrophils in the spleen of $APC^{Min/+}$ and WT mice are shown. (b-d) The number of indicated cell populations was assessed by FACS analysis. NK cells were defined as CD3- DX5+ NK1.1+; inflammatory monocytes were

defined as CD3-Ly6C+ CD11b+; DC were stained by using α-CD11c and α-CD11b; B cells were defined as CD3-, CD19+ B220+. Shown are the results of at least two independent experiments with 3 to 5 mice/group. Significance was calculated by using 2-way Anova with Bonferroni post-test (ns= not significant; * $p< 0.05$; ** $p< 0.01$; *** $p<0.001$).

Supplementary Figure 3

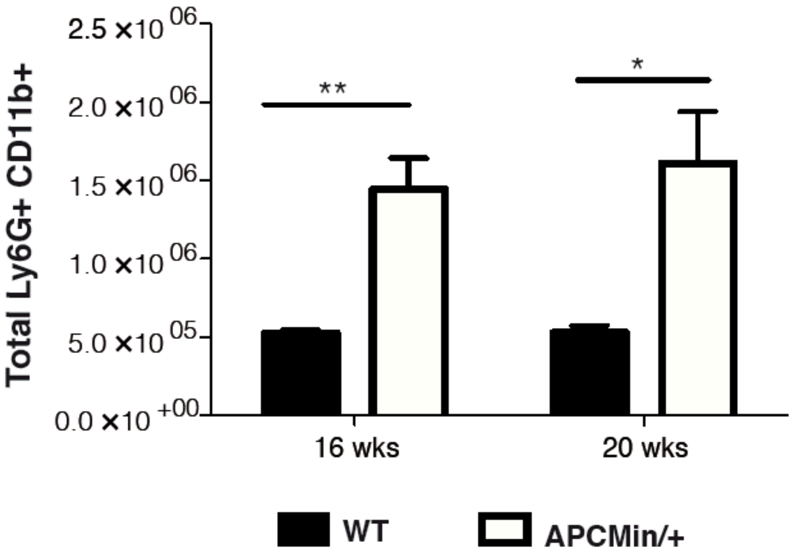

Absolute number of peripheral blood neutrophils

APC$^{Min/+}$ mice and WT littermates were sacrificed at 16 and 20 weeks of age, 1 ml of blood was collected by cardiac puncture and Ly6G microbeads were used to purify total peripheral blood neutrophils. Shown are the results of five independent experiments with 3 mice/group. Significance was calculated by using unpaired T-test (* $p < 0.05$; ** $p < 0.01$).

Supplementary Figure 4

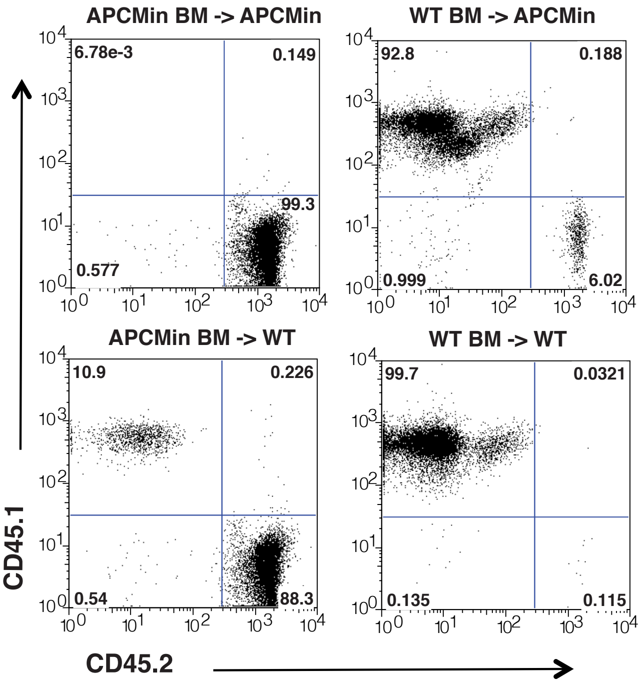

Successful reconstitution of chimeric mice after BM transplantation

1 month after BM transplantation with $APC^{Min/+}$ (CD45.2+) or WT (CD45.1+) untouched Lin- cells, successful reconstitution in chimeric mice was checked by staining for congenic markers in the blood obtained from the tail vein. Shown are representative FACS plots for each group of mice.

Supplementary Figure 5

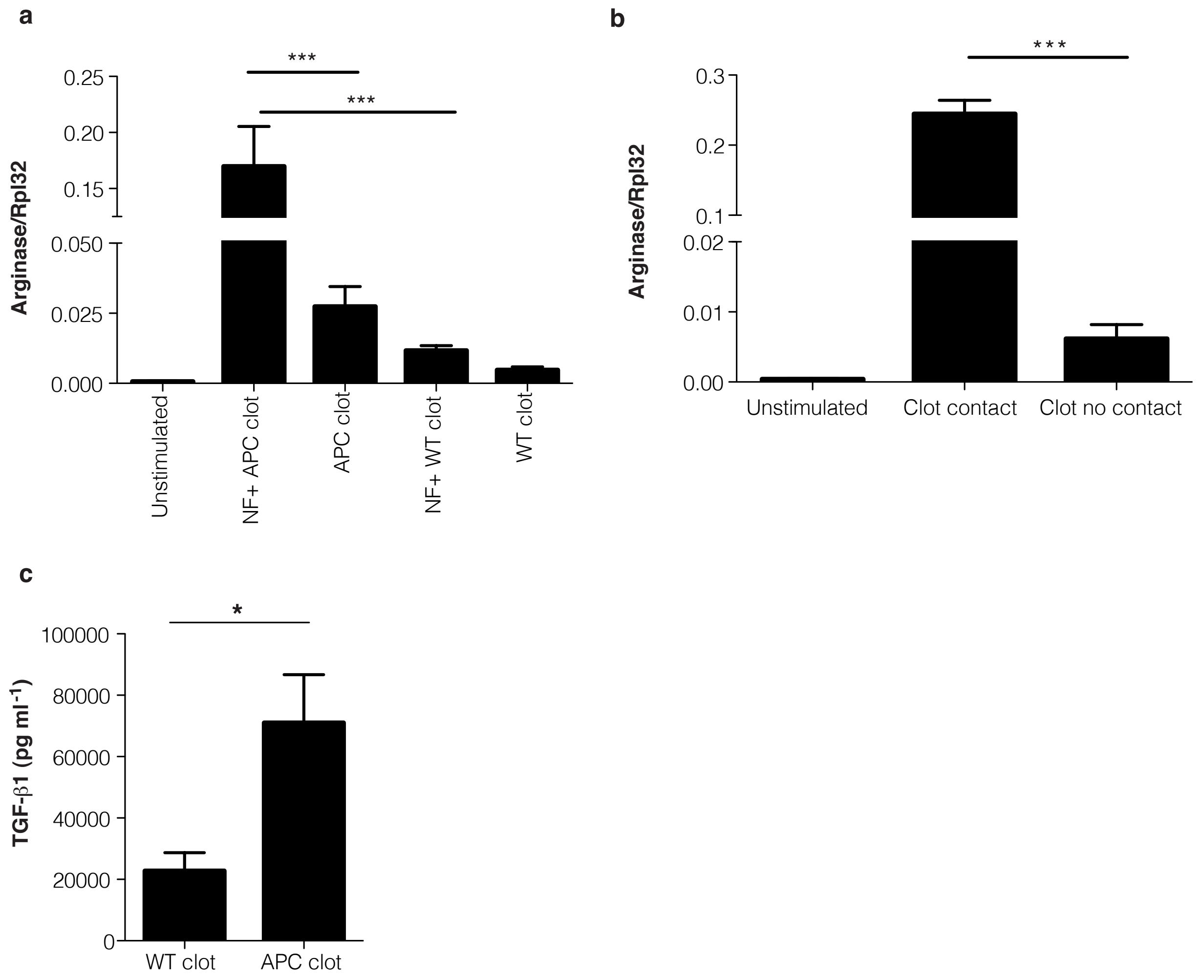

Blood clot from APC$^{Min/+}$ mice induce arginase expression in neutrophils in a contact-dependent manner and produce TGF-β1

(a) Neutrophils purified from the BM of 12 week old WT mice (CD45.1+) were stimulated with blood clots of 16 week old APC$^{Min/+}$ mice (CD45.2+). After 12 hours co-colture, BM-derived clot cells and neutrophils were magnetically separated based on congenic markers and used for RNA extraction to assess Arginase expression. (b) Arginase expression by real time PCR was assessed in BM-derived neutrophils left unstimulated or after co-colture with blood clots, which were either in contact with neutrophils or placed on a 0,4μm impermeable membrane (no contact). After 12 hours, arginase expression in neutrophils was evaluated by real time PCR. Shown are the results of three independent experiments. Significance was calculated by using 1-way Anova with Bonferroni post-test (*** $p<0.001$; * $p<0.05$). (c) After overnight culture, supernatants of blood clots from 16-

20 week old WT and $APC^{Min/+}$ mice were used to measure TGF-β1 by ELISA. Shown are the results of two independent experiments with 3 to 5 mice/group. Significance was calculated by using unpaired T-test (* $p < 0.05$).

Supplementary Figure 6

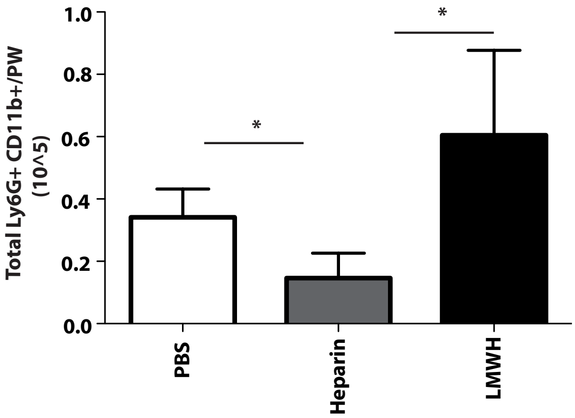

LMWH in vivo does not interfere with neutrophil migration

3 WT mice 12 week old were s.c. treated twice with PBS, 30IU of heparin or 30IU LMWH before i.p. injection of 3% thioglycollate. Treatment with PBS, heparin or LMWH was repeated 5 minutes before thioglycollate injection. Animals were sacrificed after 2 hours, the peritoneal wash was collected and the number of $CD3^{-}CD11b^{+}Ly6G^{+}$ neutrophils evaluated by FACS analysis. Results are pooled from two independent experiments. Significance was calculated by using 1-way Anova with Bonferroni post-test (* $p< 0.05$).

Supplementary Figure 7

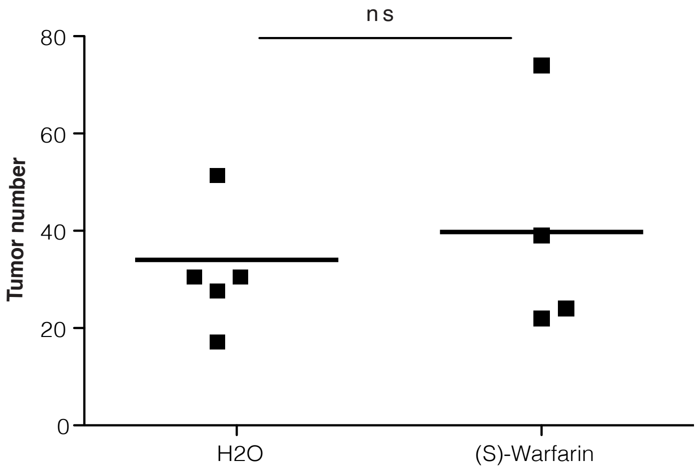

Treatment with warfarin is uneffective in reducing intestinal polyps in $APC^{Min/+}$ mice

7 week old $APC^{Min/+}$ mice were left untreated or treated with 2,5mg/ml of (S)-warfarin in the drinking water for 12 weeks. Intestinal tumor number was determined at the end of the experiment. N=4-5 mice/group. Results are representative of two independent experiments. Significance was calculated by using unpaired T-test (ns= not significant).

Supplementary Figure 8

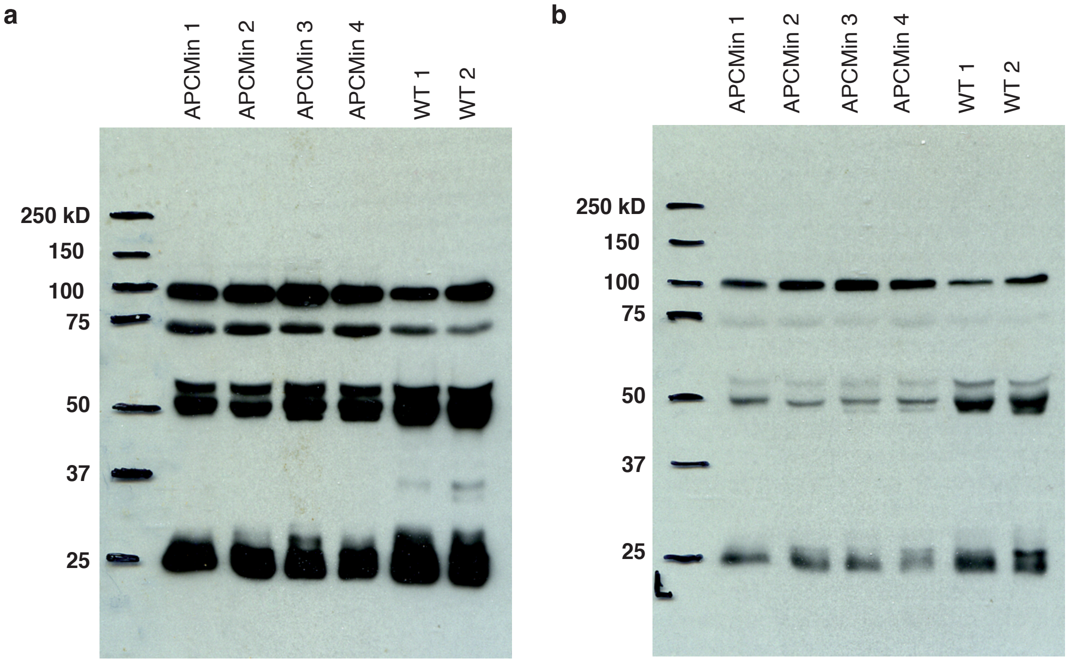

Complement activation via the alternative pathway

Plasma from 16 week old APC$^{Min/+,}$ and WT mice was used to assess complement activation by measuring Factor B (93 KDa) and Factor Bb (65KDa) by western blot. Shown are the uncropped scans of the western blot and the molecular weight marker used after (a) 1minute and (b) after 10 seconds exposure. As molecular weight marker we used the Dual Color Standard. This marker consists of highly purified recombinant proteins covalently stained with multicolored dyes, that do not develop on the film during the chemiluminescent reaction. Therefore, the band location on the film was highlighted by an ink marker.

Supplementary Figure 9

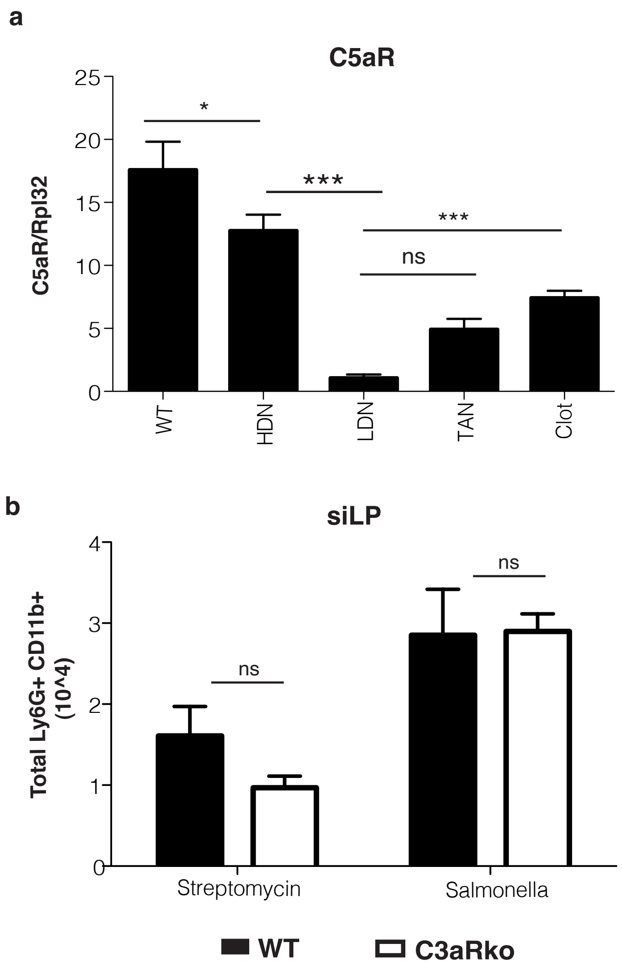

C5aR levels in neutrophils and effect of absence of C3aR signaling on neutrophil migration

(a) C5aR levels were measured by qPCR in neutrophils from 16 week old WT mice and in $APC^{Min/+}$ HDN, LDN, TAN and clot-treated BM neutrophils. Significance was calculated by using 1-way Anova with Bonferroni post-test (ns= not significant; *p>0.05; ***p< 0.001).

(b) 12 week old C3aR-/- and WT littermates were s.c. injected with 2mg/ml streptomycin. After 24 hours mice were fed by oral gavage with 500.000 CFU of Salmonella or with vehicle and were sacrificed 20 hours later. Small intestines were harvested, lamina propria cells isolated and the number of $CD45.2^{+}CD3^{-}CD11b^{+}Ly6G^{+}$ neutrophils was determined by FACS analysis. Significance was calculated by using 2-way Anova with Bonferroni post-test (ns= not significant; * p< 0.05; ** p< 0.01). Results are representative of two independent experiments with 3-5 mice/group.

Supplementary Figure 10

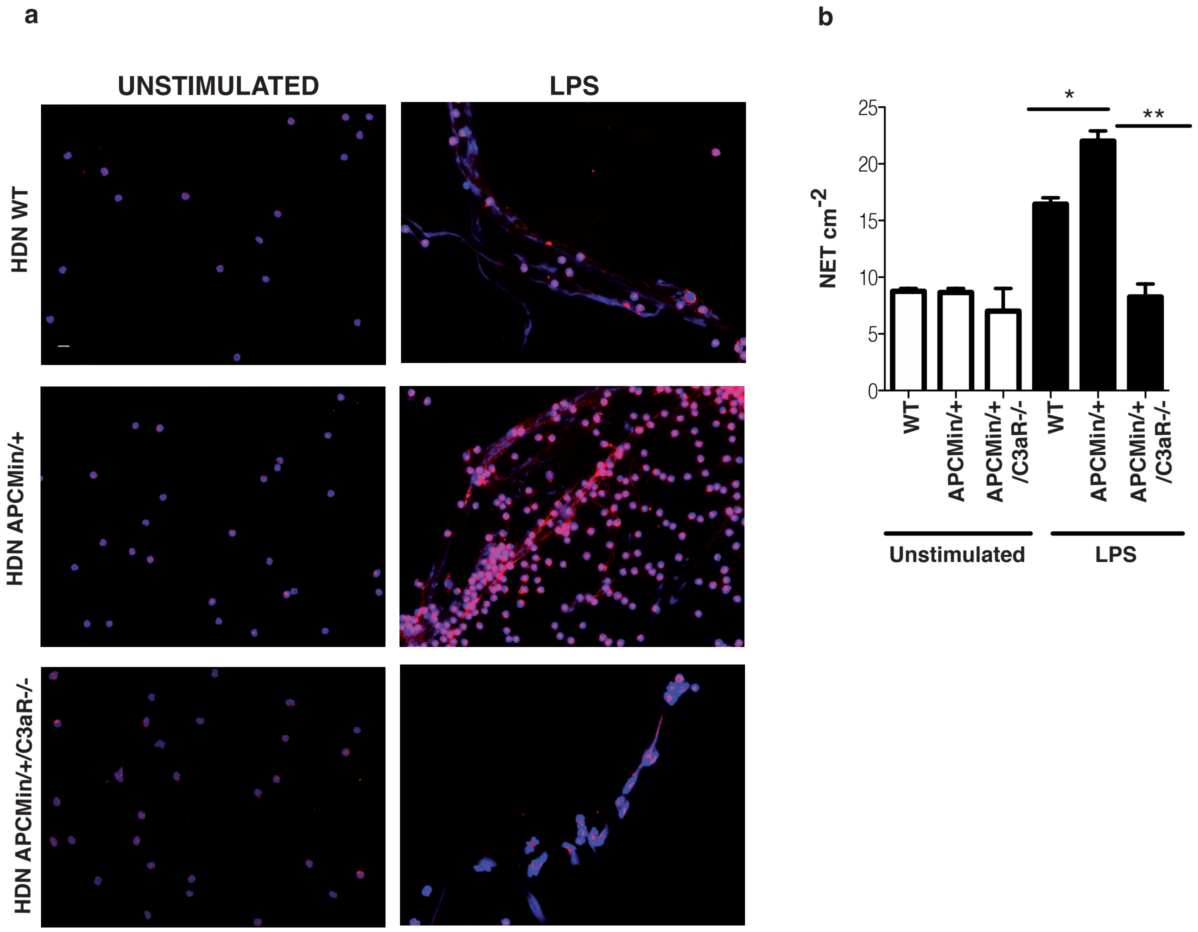

Absence of C3aR reduced LPS-induced NET formation in HDN of $APC^{Min/+}$/C3aR-/- mice

(a-b) HDN were purified from 16 week old $APC^{Min/+}$ and $APC^{Min/+}$/C3aR-/- and WT mice and left unstimulated or stimulated for 3h with LPS (10µg/ml). NETs were stained with DAPI (blue) and MPO (red) and visualized by widefield fluorescence microscopy. In (a) representative merged pictures are shown for unstimulated (left panels) and stimulated (right panels) HDN of WT, $APC^{Min/+}$ and $APC^{Min/+}$/C3aR-/- mice. Magnification 20x. Scale bar=40µm. (b) For quantification, NETs were counted on the whole slide and expressed as NET cm^{-2}. Results are pooled from two independent experiments with 2-3 mice/group. Significance was calculated by using 1-way ANOVA with Bonferroni post-test (* $p< 0.05$; ** $p< 0.01$).

Supplementary Table 1. Characteristics of CRC patients

	Categories	N	Median (q1, q3) / %
**Age median (q1, q3)**		466	64 (56, 71)
**Gender**	F	208	45%
	M	258	55%
**Body mass index (BMI) median (q1, q3)**		427	25 (23, 27)
**Cancer subtype**	Colon	249	53%
	Rectum	217	47%
**Tumor burden median (q1, q3)**		441	4 (3, 6)
	<4 cm	192	41%
	≥4cm	247	53%
	NA	27	6%
**Tumor stage (pT)**	0	2	0%
	1	20	4%
	2	99	21%
	3	298	64%
	4	45	10%
	NA	2	0%
**Grade**	G1	31	7%
	G2	304	65%
	G3	66	14%
	NA	65	14%
**Lymph-node status**	Negative	224	48%
	positive	234	50%
	NA	8	2%

Supplementary Table 2. Median values and interquartile ranges in patients with CRC

Tumor stage	Patients (n)	INR (0.8-1.2)	NLR	Neutrophils (40-74%)
pT0-pT2	122	1.03 (0.98-1.06)	2.9 (2.1-3.8)	66 (60-71)
pT3-pT4	344	1.04 (1.00-1.09)	3.1 (2.2-4.4)	67 (61-73.8)

Supplementary Table 3. Factors associated with pT>2

Variables	Categories	OR	Low	Up	P-values
**Age**		1.01	0.99	1.03	0.461
**Subtype**	Colon vs rectum	2.46	1.57	3.84	<0.0001
**Neutrophils**		0.99	0.96	1.02	0.368
**NLR**	>5 vs ≤5	3.70	1.60	8.57	0.002
**INR**		4.26	0.41	44.54	0.226
